# Modularity of RBC hitchhiking with polymeric nanoparticles: testing the limits of non-covalent adsorption

**DOI:** 10.1186/s12951-022-01544-0

**Published:** 2022-07-16

**Authors:** Vincent Lenders, Remei Escudero, Xanthippi Koutsoumpou, Laura Armengol Álvarez, Jef Rozenski, Stefaan J. Soenen, Zongmin Zhao, Samir Mitragotri, Pieter Baatsen, Karel Allegaert, Jaan Toelen, Bella B. Manshian

**Affiliations:** 1 Translational Cell and Tissue Research Unit, Department of Imaging and Pathology, KU Leuven, Herestraat 49, B3000 Louvain, Belgium; 2 Medicinal Chemistry, Rega Institute for Medical Research, Department of Pharmaceutical and Pharmacological Sciences, KU Leuven, B3000 Louvain, Belgium; 3 NanoHealth and Optical Imaging Group, Department of Imaging and Pathology, KU Leuven, Herestraat 49, B3000 Louvain, Belgium; 4 Department of Pharmaceutical Sciences, College of Pharmacy, University of Illinois at Chicago, Chicago, IL 60612 USA; 5 University of Illinois Cancer Center, Chicago, IL 60612 USA; 6 John A. Paulson School of Engineering and Applied Sciences, Harvard University, Cambridge, MA02138 USA; 7 Wyss Institute of Biologically Inspired Engineering, Harvard University, Boston, MA02115 USA; 8 VIB-KU Leuven Center for Brain and Disease Research Electron Microscopy Platform of the VIB Bioimaging Core, Louvain, Belgium; 9 Department of Neurosciences, Leuven Brain Institute, KU Leuven, B3000 Louvain, Belgium; 10 Department of Hospital Pharmacy, Erasmus MC University Medical Center, 3015, CN Rotterdam, the Netherlands; 11 Clinical Pharmacology and Pharmacotherapy, Department of Pharmaceutical and Pharmacological Sciences, KU Leuven, B3000 Louvain, Belgium; 12 Leuven Child and Youth Institute, KU Leuven, 3000 Leuven, Belgium; 13 Woman and Child, Department of Development and Regeneration, KU Leuven, 3000 Louvain, Belgium; 14 Department of Pediatrics, University Hospitals Leuven, 3000 Louvain, Belgium

**Keywords:** Drug delivery, Polymeric nanoparticles, Red blood cells, Non-covalent adsorption, Surface properties

## Abstract

Red blood cell (RBC) hitchhiking has great potential in enhancing drug therapy, by improving targeting and reducing rapid clearance of nanoparticles (NPs). However, to improve the potential for clinical translation of RBC hitchhiking, a more thorough understanding of the RBC-NP interface is needed. Here, we evaluate the effects of NP surface parameters on the success and biocompatibility of NP adsorption to extracted RBCs from various species. Major differences in RBC characteristics between rabbit, mouse and human were proven to significantly impact NP adsorption outcomes. Additionally, the effects of NP design parameters, including NP hydrophobicity, zeta potential, surfactant concentration and drug encapsulation, on RBC hitchhiking are investigated. Our studies demonstrate the importance of electrostatic interactions in balancing NP adsorption success and biocompatibility. We further investigated the effect of varying the anti-coagulant used for blood storage. The results presented here offer new insights into the parameters that impact NP adsorption on RBCs that will assist researchers in experimental design choices for using RBC hitchhiking as drug delivery strategy.

**Supplementary Information:**

The online version contains supplementary material available at 10.1186/s12951-022-01544-0.

## Introduction

Over the past years, with the surge in research efforts to modulate the pharmacokinetics and pharmacodynamics of therapeutics, red blood cells (RBCs) have attained considerable attention as a promising drug delivery system [1–3]. RBCs are of particular interest because they offer suitable biological properties, are easily accessible and obtainable in high numbers, and they have a long circulation lifespan [4]. Multiple techniques for delivering cargoes through RBCs have been developed; cargoes can be encapsulated within the RBCs by means of hypotonic treatment [5], cargoes can be coated with RBC membranes, leading to RBC-mimicking particles [6], or cargoes can be attached onto the RBC surface. Attachment to RBCs has the advantage of not only inflicting the least stress to the cells, but also fully leveraging the high surface-to-volume ratio of RBCs, which has been proven to be beneficial for the delivery of proteins [7, 8] and enzymes [9]. Additionally, coupling nanoparticles (NPs) to RBCs overcomes current translational shortcomings of NP-based therapy [10–12], by reducing clearance by the reticulo-endothelial system (RES), increasing blood circulation time and reducing unwanted adverse side effects [13].

*Ex vivo* surface binding of NPs on RBCs can be achieved by either specific interactions, *e.g*. through antibody targeting [14] or by non-specific adsorption. Regardless of mode of attachment, these strategies are referred to as ‘RBC hitchhiking’ and represent the simplest, yet successful methods of RBC surface attachment [15, 16]. NPs have been shown to adsorb on RBCs via hydrophobic interactions, electrostatic forces, hydrogen bonding and Van der Waals forces. These reversibly attached NPs improve in vivo targeting to capillary dense organs, where the coupled NPs are released from the cell surface by capillary shear forces [17]. RBC hitchhiking is particularly of interest for improved targeting towards the lungs, due to a high local blood flux and a large pulmonary endothelial surface, although other organs, such as the heart and the brain, have also shown targeting potential [18].

To ensure effective translation of RBC hitchhiking into the clinic, gaining insights into sensitivity to parameters such as variations in RBC characteristics and NP properties is crucial to establishing the versatility of RBCs as a drug delivery platform. Eventually, understanding the NP-biomolecule interaction interface is thought to help reduce animal testing and hasten clinical trials [19]. While some previous reports have evaluated the biocompatibility of nanomaterials with RBCs [20, 21], an in depth understanding of the modularity, and key influential parameters that dictate the formation of RBC-NP-complex are lacking.

**Fig. 1:**
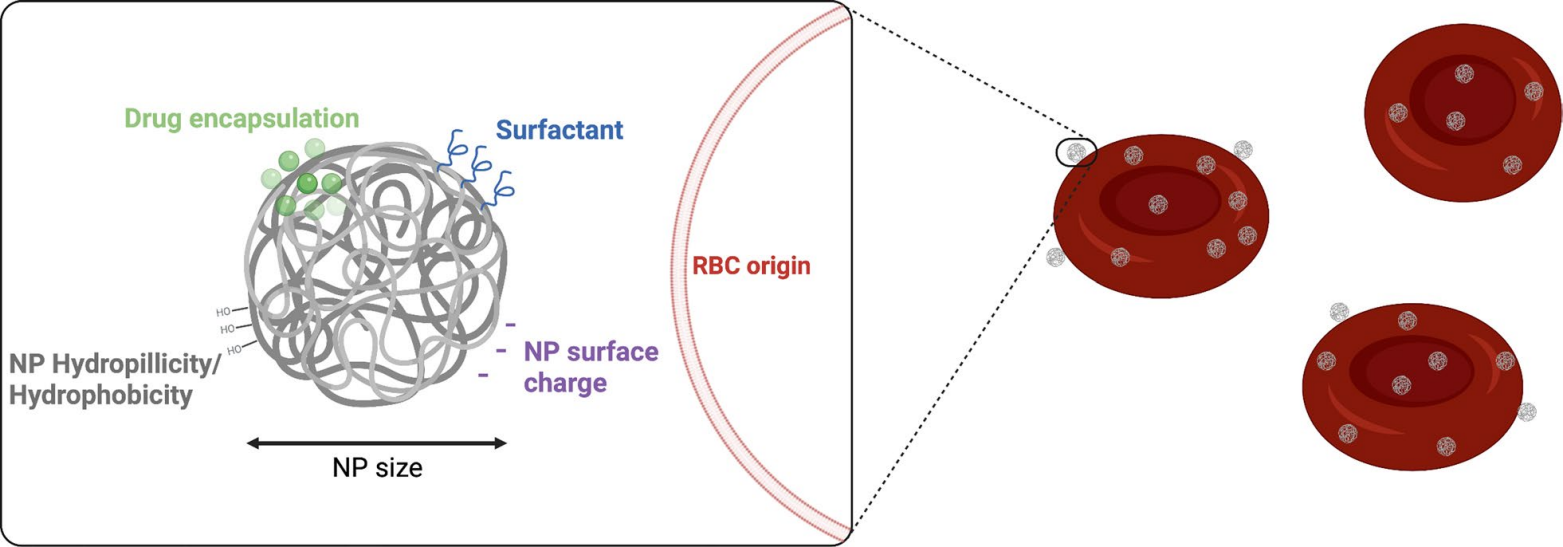
Overview of parameters influencing the adsorption of PLGA NPs to RBCs

In this study, we evaluated different parameters involved in non-covalent NP RBC adsorption with respect to adsorption efficacy and biocompatibility (Fig. 1). Specifically, we evaluated the effect of different RBC origins, NP surface hydrophobicity, NP surface charge and drug encapsulation and illustrated the need for optimization of each of these elements for a given application. The effect of NP size was not investigated in this study, but has already been illustrated in other reports [22, 23]. Hereby, we suggest a framework of parameters that should be accounted for when performing non-covalent NP adsorption on extracted RBCs for drug delivery purposes. Finally, this study highlights the limits of RBC hitchhiking with the current understanding of the NP-RBC interface.

## Materials and methods

Animal blood collection was approved by the Ethics committee for Animal Experimentation of KU Leuven (P074/2019 and P219/2018) and performed according to current animal welfare guidelines. Human blood donation was approved by the Ethic Committee Research UZ/KU Leuven (S65268) and donated by healthy volunteers.

### Nanoparticle synthesis and characterization

Polymeric nanoparticles were synthesized using the nanoprecipitation method. Shortly, 20 mg PLGA and 200 µg Cy7.5 was dissolved in 1 mL acetone and vortexed to ensure full and homogenous dissolvement. The organic phase was then added, dropwise, with a syringe pump (FisherBrand™ KDS100 Legacy Syringe pump) to 10 mL of 1.5% PVA MQ solution, under constant stirring. The solution was kept overnight, under constant stirring, to allow evaporation of the organic phase.

Nanoparticles were then washed 3 times at 12.000 rpm with Milli-Q water and resuspended in either MQ water for characterization or PBS for RBC coupling. Nanoparticles were characterized using dynamic light scattering (Malvern Panalytical, Zetasizer Nano ZSP, United Kingdom) and the hydrodynamic size, PDI and zeta potential were measured. Nanoparticle concentration (number of particles/mL) was determined using nanoparticle tracking analysis (Malvern NanoSight LM10, Malvern Panalytical, United Kingdom). For PEI-NPs, 0.5 mg polyethylenimine was added to the water phase. Further steps were similar as for plain NPs. For drug-loaded NPs, 10 mg dexamethasone or 0.5 mg paclitaxel was added to the organic phase consisting of 20 mg PLGA and 200 µg Cy7.5 dissolved in 1.5 mL acetone. Further steps were similar as for plain NPs.

### Blood collection

Rabbit (New Zealand White-Flemish Giant Hybrid) blood was collected using heparinized syringes (BD vacutainer) via aspiration of the ear artery, after sedation with ketamine/xylazine. Mouse blood was collected from C57BL/6 mice via cardiac puncture performed under anesthesia (2% isoflurane), using heparinized syringes (BD vacutainer). Human blood was collected from volunteers with blood type O negative (O-).

All blood samples were collected in blood storage containers with lithium heparin 75 USP units (BD Vacutainer), unless indicated otherwise.

### RBC isolation

For each experiment, freshly obtained blood was used. Whole blood was centrifuged at 1000* g* for 10 min at 4 °C to separate the plasma and serum from the RBCs. Plasma was discarded, and the isolated RBCs were washed three times with 10 ml cold PBS by centrifugation at 650* g* for 15 min at 4 °C. RBCs were resuspended at a final concentration of 10% hematocrit in PBS.

### Coupling of nanoparticles to RBCs

Different RBC:NP ratios (1:50, 1:100, 1:500 and 1:1000) were tested. Relevant ratios were chosen based on previous works [15, 16]. Ratios were calculated based on nanoparticle concentration and RBC count. Equal volumes (150 ul) of RBC solution and NP suspension were mixed in Axygen 1.5-ml Self-Standing Screw Cap Tubes mixed by pipetting and inversion. The tubes were then rotated on a tube revolver (Thermo Fisher Scientific) at 12 rpm for 30 min. The coupled RBCs were then pelleted by centrifugation at 100* g* for 5 min at 4 °C and the pellet was washed again with 1 ml of cold PBS to remove loosely bound NPs. The supernatant was analyzed for hemoglobin presence (absorbance read at 550 nm) with a plate reader (SpectraMax iD3, Molecular Devices), to quantify RBC hemolysis during coupling. The pelleted, coupled RBCs were finally resuspended at 10% hematocrit in PBS.

### Scanning Electron Microscopy

Nanoparticles and coupled RBCs were characterized using scanning electron microscopy. Nanoparticle samples were vacuum dried in a desiccator before imaging. RBC samples were fixed in 2.5% glutaraldehyde solution and dehydrated with a graded series of ethanol. Subsequently, samples were chemically dried using hexamethyldisilazane and coated with 8 nm chromium with a Leica ACE600 coating machine (Leica, Vienna, CH) and imaged in a Zeiss Sigma FESEM (Zeiss, Oberkochen, DE) at 2 kV accelerating voltage.

### Quantification of coupling efficiency

Nanoparticle coupling onto the RBCs was verified by fluorescence measurements. For coupling efficiency determination, 25 μl of RBCs was lysed using 275 MQ water and was analyzed by quantifying the intensity of the Cy7.5 fluorescent probe using a plate reader (SpectraMax iD3, Molecular Devices).

### Quantification of NP-carrying RBC population

The percentage of RBCs carrying NPs was determined for all RBC:NP ratios using image based flow cytometry (Amnis Imagestream Mark II) using the Cy7.5 fluorescence probe. Additionally, Imagestream allowed image-based RBC morphology and quality evaluation.

### Agglutination assay

RBCs and RBC-NP samples of 1% hematocrit were transferred to a U-shaped 96-well plate and incubated overnight at 37 °C. The plates were then visually assessed for agglutination and plates were imaged with a phone camera.

### Quantification of dexamethasone encapsulation

Optimal initial DEX content was determined by measuring the encapsulation efficiency (EE%) of plain PLGA NPs synthesized with 2, 5 or 10 mg initial DEX content in the organic phase. EE% was determined by chromatographic separation using HPLC. Additional details can be found in the Supporting Information.

### Statistical analysis

All data is presented as means ± SEM. Comparison between two groups was performed using unpaired two-tailed Student’s *t* test. Comparisons among multiple groups was performed using a 2-way analysis of variance (ANOVA) with a Tukey or Sidak adjustment for multiple testing. All statistical analyses were carried out using GraphPad Prism 9 software.

## Results and discussion

### Effect of RBC characteristics on NP adsorption

RBC hitchhiking has been described in different preclinical studies, using RBCs from different species [13, 15, 16]. However, as RBC characteristics vary across species, inherent issues might complicate RBC hitchhiking in different models that can pose challenges to use this system as a generic platform. Therefore, it is of paramount importance to understand the effect of varying RBC origins on NP adsorption. To this end, we evaluated the adsorption of poly(lactic-co-glycolic acid) (PLGA) NPs on 3 different RBC types: human, mouse and rabbit. Mouse and rabbit RBCs were chosen for their specific model translationability in respiratory oncology [24] and perinatal disorders [25], respectively, while human RBCs were used for their translational potential.

**Fig. 2:**
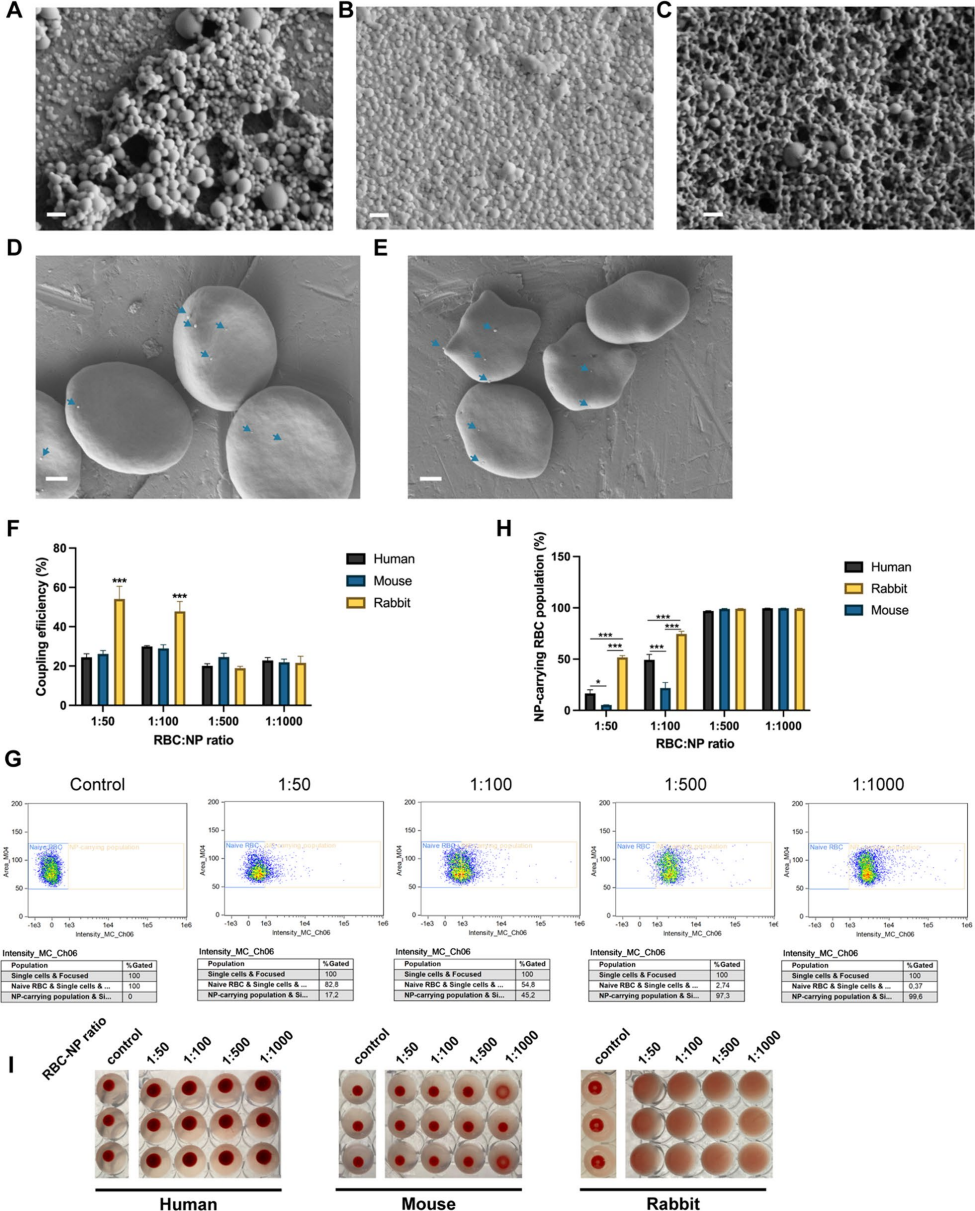
Adsorption of PLGA NPs to human, mouse and rabbit RBCs. Scanning electron microscopy image of **a** plain PLGA NPs, **b** DEX-PLGA NPs and **c** PEI-PLGA NPs. Scale bars indicate 400 nm. **d** Scanning electron microscopy images representing PLGA-NP adsorption onto human RBCs. Scale bars indicate 1 µm. **e** Scanning electron microscopy images representing PLGA-NP adsorption onto mouse RBCs. Scale bars indicate 1 µm. **f** Coupling efficiency of PLGA NPs onto human, mouse and rabbit RBCs at different RBC:NP incubation ratios. **g** Representative scatter plots of flow cytometry analysis, here shown for PLGA65:35 NPs coupled to human RBCs at different ratios. **h** Percentages of RBC populations carrying NPs. **i** Agglutination results of human, mouse and rabbit RBCs at different RBC:NP incubation ratios (n = 3). NPs used were PLGA65:35 synthesized with 1.5% PVA. All data are presented as mean ± SEM (n = 3). The number of asterisks indicate the level of significance (two-way ANOVA with Tukey multiple testing), where: *p < 0.05, **p < 0.01 and ***p < 0.001

Fluorescent PLGA NPs were synthesized using a nanoprecipitation method [15]; NPs were found to have an average hydrodynamic size of 223.1 ± 7.3 nm and an average zeta potential of − 25.9 ± 0.2 mV (Table 1, Additional file 1: Fig. S10). Further characterization using scanning electron microscopy (Fig. 2A) revealed the spherical shape and monodispersity of these NPs. After incubation of the PLGA NPs with RBCs at different RBC:NP ratios, the NP coupling success was verified by scanning electron microscopy (Fig. 2D, E), which revealed successful NP adsorption on RBCs. NP coupling was also quantitatively evaluated through fluorescence measurements using Cyanine 7.5 (Cy7.5) as a probe that was encapsulated within the NPs. The coupling efficiency results showed no difference between mouse and human RBCs, regardless of the RBC:NP incubation ratio (Fig. 2F). However, a clear difference for rabbit RBCs was noticed, with up to two-fold increase of the coupling efficiency at the lowest incubation ratios compared to mouse and human RBCs. Similar trends were detected when quantifying the percentage of RBCs carrying NPs (Fig. 2G, H). At incubation ratios above 1:500, NP binding to RBCs was saturated (NP-carrying population > 96.58%), regardless of RBC type. However, at ratios below 1:500, differences in the percentage of RBCs that carry NPs were noticed across different RBC types, with rabbit RBCs showing the highest NP-carrying population at ratios of 1:50 and 1:100, 51.63 ± 3.10 % and 74.58 ± 4.53 % respectively, while mouse RBCs showed the lowest NP-carrying population, 5.29 ± 0.33 % and 21.75 ± 9.36 % respectively.

**Table 1:** Characteristics of the PLGA nanoparticles used in this study

NP	Hydrodynamic size (nm)	Zeta potential (mV)	PDI	NP concentration (particles/mL)
PLGA65:35 1.5%PVA	223.07 ± 7.34	− 25.93 ± 0.15	0.223 ± 0.017	2.85 ± 0.08E11
PLGA50:50 1.5%PVA	194.57 ± 3.40	− 25.40 ± 0.44	0.190 ± 0.004	1.94 ± 0.07E11
PEI-PLGA65:35 1.5%PVA	302.37 ± 5.17	17.27 ± 0.35	0.273 ± 0.012	2.56 ± 0.04E11
PLGA65:35 3%PVA	257.27 ± 4.02	− 20.03 ± 0.55	0.203 ± 0.023	3.07 ± 0.04E11
PEI-PLGA65:35 3%PVA	282.13 ± 0.92	20.77 ± 0.90	0.218 ± 0.008	2.52 ± 0.11E11
DEX-PLGA65:35 1.5%PVA	212.57 ± 2.75	− 29.83 ± 0.60	0.179 ± 0.025	3.69 ± 0.04E11
PTX-PLGA65:35 1.5%PVA	276.43 ± 9.06	− 27.93 ± 0.29	0.297 ± 0.042	2.53 ± 0.04E11

*PVA* poly(vinyl alcohol); *PEI* polyethylenimine; *PDI* poly dispersity index; *DEX* dexamethasone; *PTX* paclitaxel

The differences in adsorption success for the RBC types can be attributed to their unique cellular characteristics that influence the balance of non-covalent interactions (hydrophobic interactions, electrostatic forces, hydrogen bonding and Van der Waals forces) steering spontaneous NP adsorption [17]. Such variations include the content of sialic acid on RBC membrane, with rabbit RBCs possessing a lower content as compared to human and mouse RBCs [26]. Sialic acid not only impacts the survival time and structural integrity of the cells, but leads to a lower negative charge of the cell membrane. As PLGA nanoparticles have a negative surface charge, the resulting repulsive electrostatic forces will be higher for RBCs with a higher sialic acid content, which most likely is the cause of the lower NP coupling efficiencies for the mouse and human RBCs as compared to rabbit RBCs. Rabbit RBCs, thus, serve as a more favorable vehicle to facilitate spontaneous adsorption of PLGA NPs. Note that at higher NP:RBC ratios, the difference in NP coupling capability is masked by (over)saturation of the cell surface, resulting in similar efficiencies regardless of RBC origins.

Other RBC variations include morphological differences such as size of RBCs with, for example, mouse RBCs being significantly smaller than human RBCs [27]. Relative sizes of NPs have been shown to influence the adsorption of NPs onto RBCs [23], which might explain the smaller NP-carrying mouse RBC population. Since the coupling efficiencies for mouse and human RBCs were similar, the lower NP-carrying mouse RBC population indicates a different distribution of the NPs across the RBC population. Variations in PLGA NP coupling success can also be expected for RBCs from other origins, as previously reported for nanogel adsorption on rat and pig RBCs [18].

Apart from NP adsorption success, it is of interest to evaluate any differing NP incompatibility issues when varying RBC origin. For this purpose, hemolysis induced by NP adsorption was quantified by measuring the release of hemoglobin (Additional file 1: Fig. S17). Similar hemolysis profiles were observed for all RBC types, with increased hemolysis percentages starting from a RBC:NP incubation ratio of 1:500. Standard agglutination assays performed in U-shaped well-plates showed no visible agglutination for human and mouse RBCs, in agreement with previous reports on the interaction of polymeric NPs (600nm) with human RBCs [28]. However, remarkable aggregation of rabbit RBCs could be seen after the adsorption of PLGA NPs, even at the lowest incubation ratios (Fig. 2I).

Despite that rabbit RBCs appeared to have a high potential as NP hitchhiking vehicles, the NPs act as linkers and interfere with normal stacking of the RBCs. This PLGA NP linking effect was absent for the more negatively-charged mouse and human RBCs, most likely owing to the higher electrostatic repulsion between the NPs and the cells. While PLGA NPs as such have a long known history of safety in preclinical and clinical applications [29, 30], especially compared to inorganic NPs [31, 32], compatibility of NPs with rabbit RBCs requires further consideration.

Our results indicate that fundamental differences in RBC origins have a major impact on NP adsorption efficacy and biocompatibility. These dependencies have significant implications in using RBC hitchhiking in various disease models. For translational *ex vivo* RBC adsorption research with new polymeric formulations, it is therefore advisable to perform comparison studies between human RBCs and the RBCs of the chosen animal model. Additionally, for highly differing RBCs origins, as in the case for rabbit RBCs, the use of complementary animal disease models is advised to enhance clinical relevance.

Finally, it should be noted that any change in experimental set-up can further influence NP adsorption, for example, the choice of the anti-coagulant used for blood storage. Upon RBC isolation from whole blood, stored in an anti-coagulant, trace amounts of the anti-coagulant can remain on the surface of the isolated cells despite repeated washes. The presence of the residual anti-coagulant might influence NP adsorption. Therefore, in order to maximize coupling efficiency of PLGA NPs to human RBCs, our results (Additional file 1: Fig. S18) showed that lithium heparin is preferred over other anti-coagulants, such as ethylenediamine tetraacetic acid (EDTA) and sodium citrate.

### Effect of NP surface characteristics on NP adsorption

#### Nanoparticle hydrophobicity and surface charge

Beside RBC properties, NP surface characteristics are expected to influence NP adsorption. We therefore evaluated the effect of different NP characteristics on the coupling efficiency and biocompatibility. Firstly, the effect of NP hydrophobicity on NP adsorption onto human RBCs was evaluated by comparing 2 PLGA types with different lactic:glycolic acid ratios (65:35 and 50:50), with a higher lactic acid content leading to more hydrophobic polymeric matrices. Lactic acid impedes hydrogen bonding with water molecules due to steric shielding of the methyl side groups. PLGA50:50 NPs were characterized to be similar in size and zeta potential as PLGA65:35 NPs (Table 1, Additional file 1: Fig. S11).

**Fig. 3:**
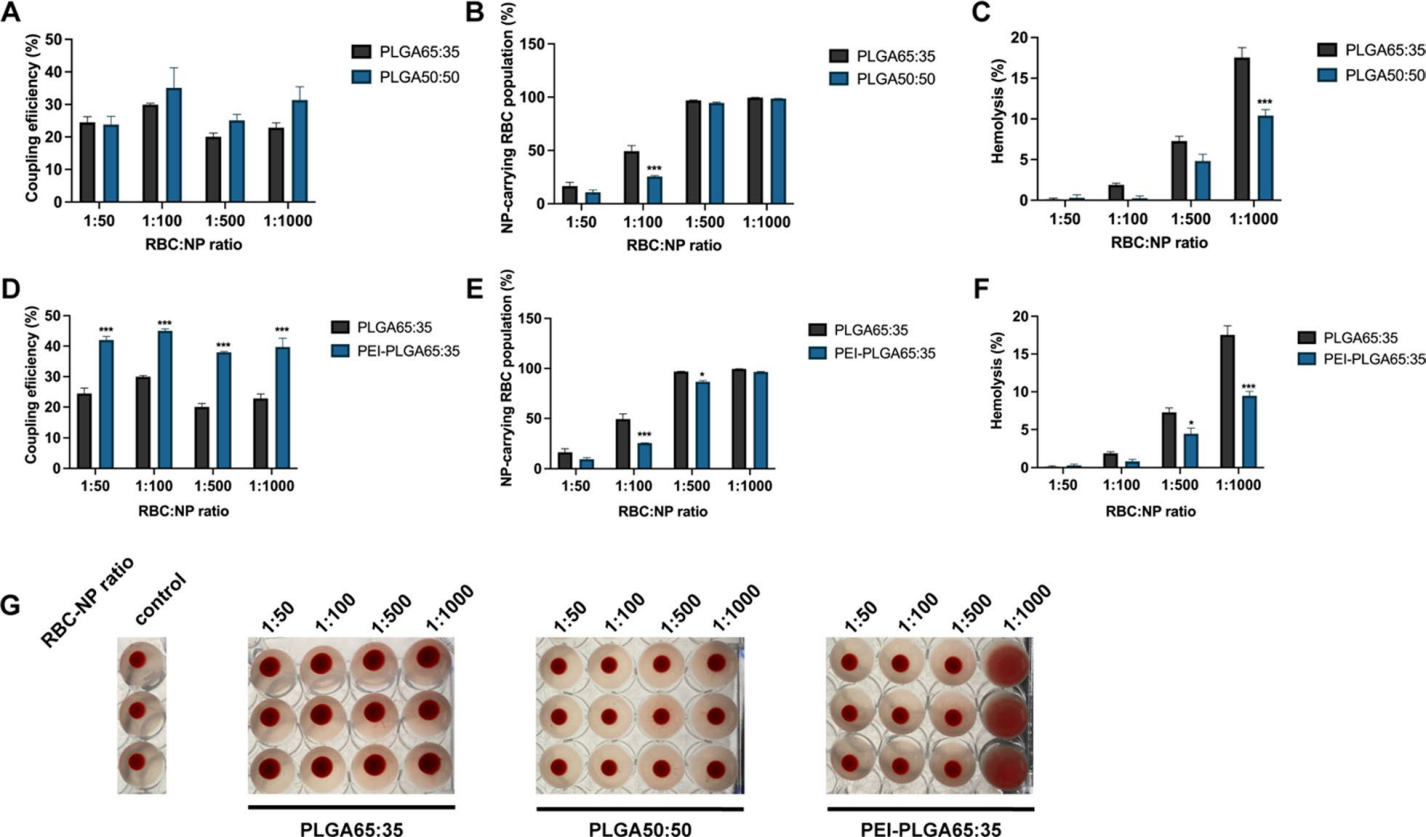
Hydrophobicity and zeta potential effect on NP adsorption to human RBCs. **a** Coupling efficiency of PLGA65:35 and PLGA50:50 NPs onto human RBCs at different RBC:NP incubation ratios. **b** Percentages of human RBC populations carrying PLGA65:35 or PLGA50:50 NPs. **c** Hemolysis percentages of human RBCs after incubation with PLGA65:35 or PLGA50:50 NPs at different RBC:NP incubation ratios. Hemolysis percentages are represented as total hemolysis subtracted by hemolysis of control cells. **d** Coupling efficiency of PLGA65:35 and PEI-PLGA65:35 nanoparticles onto human RBCs at different RBC:NP incubation ratios. **e** Percentages of human RBC populations carrying PLGA65:35 or PEI-PLGA65:35 NPs. **f** Hemolysis percentages of human RBCs after incubation with PLGA65:35 or PEI-PLGA65:35 NPs at different RBC:NP incubation ratios. Hemolysis percentages are represented as total hemolysis subtracted by hemolysis of control cells. **g** Agglutination assay of human RBCs with PLGA65:35 or PLGA50:50 NPs or PEI-PLGA65:35 NPs at different RBC:NP incubation ratios (n = 3). All data are presented as mean ± SEM (n = 3). The number of asterisks indicate the level of significance (two-way ANOVA with Sidak multiple testing adjustment), where: *p < 0.05, **p < 0.01 and ***p < 0.001

NP hydrophobicity had minimum impact on the coupling efficiency (Fig. 3A), while a slight change could be noted in the NP-carrying population (Fig. 3B), with a less homogenous distribution of PLGA50:50 NPs (25.53 ± 1.61 %) compared to PLGA65:35 NPs (49.26 ± 9.27 %) at an incubation ratio of 1:100. These results imply that the difference in the hydrophobicity of two PLGA types does not influence the non-covalent interaction balance. The lesser hydrophobic interactions for PLGA50:50 NPs are likely to be compensated by other non-covalent interactions, such as more hydrogen bonding. Next, we evaluated the influence of NP hydrophobicity on NP adsorption and biocompatibility. NP hydrophobicity had a clear influence on the level of RBC lysis induced following RBC-NP coupling. PLGA65:35 NPs led to a 1.7-fold increase in hemolysis at the highest incubation ratio (1:1000) compared to PLGA50:50 NPs, indicating that the added hydrophobic interactions for PLGA65:35 NPs increase stress to the RBCs (Fig. 3C). No alterations in blood agglutination were detected from coupling to the different PLGA types (Fig. 3G). These results imply that, despite the fact that the overall balance of non-covalent interactions for both PLGA NPs is similar, the composition and contribution of each non-covalent interaction (hydrophobic interactions, electrostatic forces, hydrogen bonding and Van der Waals forces) still impacts NP adsorption, especially in terms of NP biocompatibility.

As discussed previously, RBC origin impacts both NP coupling success and biocompatibility. Therefore, we also tested the effect of NP hydrophobicity on NP adsorption for mouse and rabbit RBCs. Our results indicate that the effect of changing hydrophobicity manifests differently depending on RBC origin. For mouse RBCs an increase in coupling efficiency was seen at the lowest 1:50 incubation ratio (Additional file 1: Fig. S19). Moreover, agglutination of RBCs at the highest PLGA50:50 NP incubation ratios (1:500 and 1:1000) was visible. This indicates an increased linking of the smaller mouse RBCs, probably due to improved hydrogen bonding. In contrast, changing NP hydrophobicity did not influence adsorption to rabbit RBCs (Additional file 1: Fig. S20), both in terms of coupling success, with similar coupling efficiency, as well as biocompatibility, with similar hemolysis induction and strong agglutination at all ratios. The lack of a (stronger) repulsive electrostatic force seems to overshadow any potential effect of changing the NP hydrophobicity, illustrating the significant contribution of electrostatic forces in the NP adsorption mechanism. Optimizing formulation design with regards to PLGA monomer ratio, can therefore, if the biocompatibility requirement is met, mainly be driven by the desired hydrolytic rate of the NPs.

To further illustrate the importance of electrostatic forces, we tested the effect of varying NP zeta potential on NP adsorption onto human RBCs, by comparing negatively charged PLGA NPs with positively charged polyethylenimine (PEI) PLGA NPs. The latter were characterized to have an average zeta potential of 17.3 ± 0.4 mV and an average size of 302.4 ± 5.2 nm (Table 1, Additional file 1: Fig. S12). In addition, NPs exhibited a spherical shape and mono-dispersity as characterized by scanning electron microscopy (Fig. 2C). Adsorption results revealed up to two-fold increase in coupling efficiency for PEI-PLGA NPs at all incubation ratio’s compared to plain PLGA NPs (Fig. 3D). This indicates that NP adsorption is facilitated by the electrostatic attraction of positively charged NPs and negatively charged red blood cells. However, this strong attraction led to a less homogeneous distribution of the particles at ratios of 1:100 (25.68 ± 0.36 %) and 1:500 (86.70 ± 2.11 %) (Fig. 3E) and linked the red blood cells at high incubation ratios, leading to agglutination (Fig. 3G). Surprisingly, even though positively charged NPs will bind more efficiently onto RBCs, the induced hemolysis was significantly less for PEI-PLGA NPs at higher incubation ratios, with, compared to plain PLGA NPs, a 1.4-fold and 1.5-fold decrease at ratio 1:500 and 1:1000, respectively (Fig. 3F). This can be explained by the greater contribution of electrostatic forces for adsorption of PEI-NPs, and the lesser influence of hydrophobic interactions, which appear to be more damaging to RBCs. For mouse RBCs, similar trends were noticed, with only slight differences in NP-carrying RBC population percentages, likely because of their smaller size (Additional file 1: Fig. S21). For rabbit RBCs, results were also similar to human RBCs, however, less pronounced, as electrostatic attraction forces were weaker for the less negatively charged rabbit RBCs than for human RBCs (Additional file 1: Fig. S22). These results indicate that formulation designs using, particularly, positively charged NPs should undergo proper biocompatibility evaluation.

#### Surfactant concentration

**Fig. 4:**
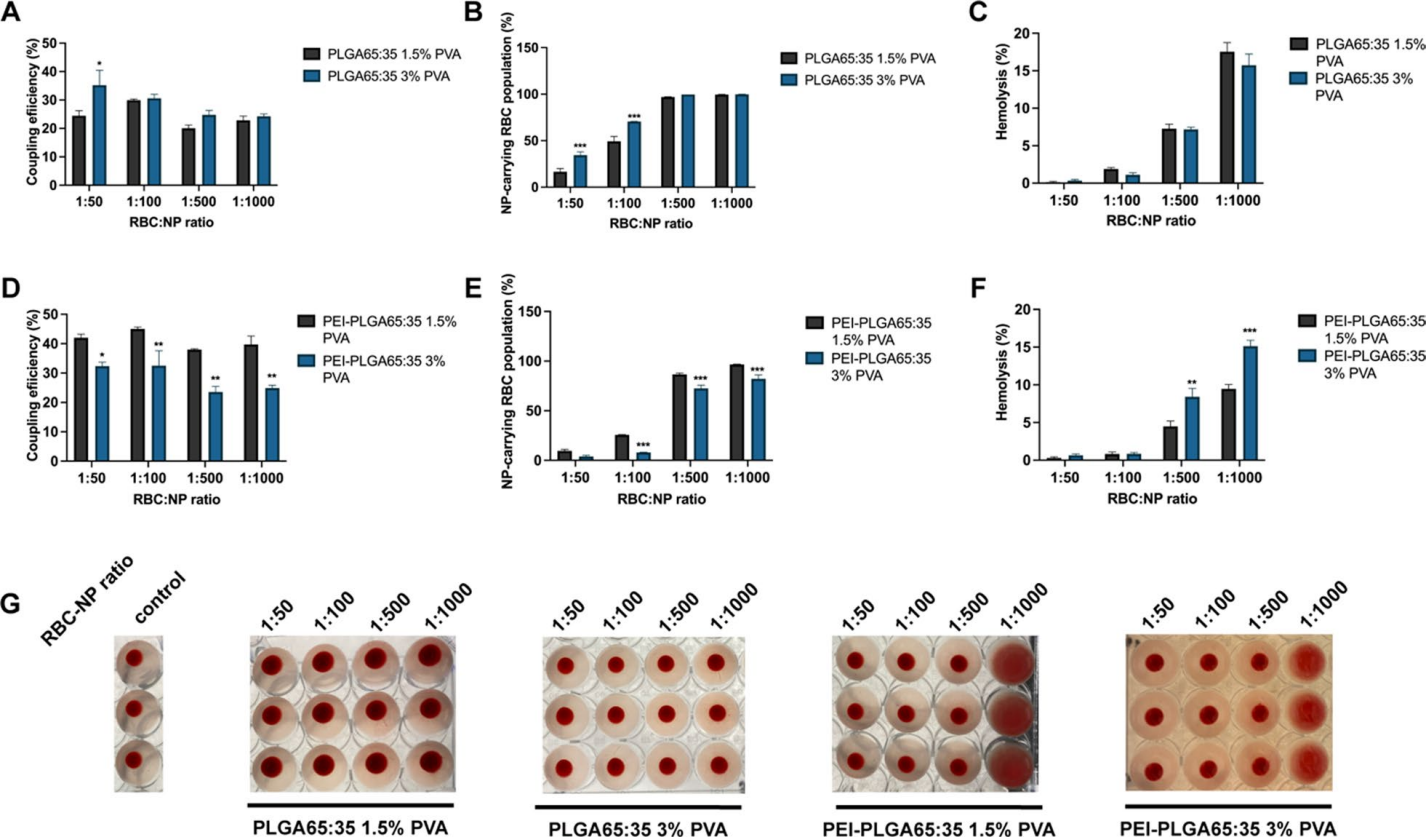
Effect of surfactant concentration on NP adsorption to human RBCs. **a** Coupling efficiency of PLGA65:35 NPs, synthesized with either 1.5% PVA or 3% PVA, onto human RBCs at different RBC:NP incubation ratios. **b** Percentages of human RBC populations carrying PLGA65:35 NPs, synthesized with either 1.5% PVA or 3% PVA. **c** Hemolysis percentages of human RBCs after incubation with PLGA65:35 NPs, synthesized with either 1.5% PVA or 3% PVA, at different RBC:NP incubation ratios. Hemolysis percentages are represented as total hemolysis subtracted by hemolysis of control cells. **d** Coupling efficiency of PEI-PLGA65:35 NPs, synthesized with either 1.5% PVA or 3% PVA, onto human RBCs at different RBC:NP incubation ratios. **e** Percentages of human RBC populations carrying at least 1 PEI-PLGA65:35 NP, synthesized with either 1.5% PVA or 3% PVA. **f** Hemolysis percentages of human RBCs after incubation with PEI-PLGA65:35 NPs, synthesized with either 1.5% PVA or 3% PVA, at different RBC:NP incubation ratios. Hemolysis percentages are represented as total hemolysis subtracted by hemolysis of control cells. **g** Agglutination assay of human RBCs with PLGA65:35 or PEI-PLGA65:35 NPs, synthesized with either 1.5% PVA or 3% PVA, at different RBC:NP incubation ratios (n = 3). All data are presented as mean ± SEM (n = 3). The number of asterisks indicate the level of significance (two-way ANOVA with Sidak multiple testing adjustment), where: *p < 0.05, **p < 0.01 and ***p < 0.001

Synthesizing PLGA nanoparticles via the nanoprecipitation method requires the use of a surfactant, in this case poly(vinyl alcohol) (PVA), to reduce coalescence of the emulsion. However, the concentration of the surfactant should be chosen carefully, because even after repeated washing steps, certain quantities of PVA remain adsorbed onto the NPs. A higher PVA concentration can lead to a higher PVA surface adsorption [33, 34]. Increased PVA adsorption will influence NP surface properties and might influence NP-RBC adsorption. In order to understand the role the surfactant might play in NP adsorption, we compared the coupling efficiency of plain PLGA and PEI-PLGA NPs that were synthesized using either 1.5% or 3% PVA. The difference in PVA concentration resulted in expected changes for plain PLGA NP characteristics [34], with 3% PVA NPs manifesting as slightly larger (257.27 ± 4.02 nm) and slightly less negatively charged (− 20.03 ± 0.55 mV) (Table 1, Additional file 1: Fig. S13). NP adsorption results for plain PLGA nanoparticles indicated only slight differences for different PVA concentrations, with a higher coupling efficiency, at the lowest RBC:NP incubation ratio (1:50), for 3% PVA PLGA NPs (35.22 ± 9.04 %) compared to 1.5%PVA PLGA NPs (24.46 ± 3.06 %) (Fig. 4A). Additionally, NP-carrying RBC population percentages were higher for 3% PVA NPs at ratios 1:50 and 1:100, 34.47 ± 6.19 % and 70.59 ± 0.32 % respectively, compared to 1.5% PVA PLGA NPs, 16.51 ± 6.25 % and 49.26 ± 9.27 respectively (Fig. 4B). Likely, this can be attributed to the slightly less negative zeta potential of 3% PVA NPs and the added hydrogen bonding facilitated by the chemical structure of PVA. However, for most RBC:NP ratios, no difference is observed when changing PVA concentration, neither in coupling success nor in biocompatibility (Fig. 4C, G), suggesting that the added hydrogen bonding and reduced electrostatic repulsion from PVA adsorption is largely balanced by reduced hydrophobic interactions due to PVA shielding of hydrophobic parts of the PLGA core. For PEI-PLGA NPs, increasing PVA concentration resulted in slightly smaller (282.13 ± 0.92 nm) and more positively charged (+ 20.77 ± 0.90 mV) NPs (Table 1 and Additional file 1: Fig. S6). Varying PVA concentration for the PEI-PLGA NPs led to a much stronger effect on NP adsorption, with 1.3, 1.4, 1.6 and 1.6-fold lower coupling efficiencies, respectively, for NPs prepared with 3% PVA, at all the tested ratios (Fig. 4D). While the NP-carrying RBC population percentages were 3.2, 1.2 and 1.2-fold lower for ratios 1:100, 1:500 and 1:1000 (Fig. 4E), and 1.9 and 1.6-fold higher hemolysis percentages at ratios 1:500 and 1:1000 (Fig. 4F).

Overall, these results indicate that when the surfactant is the only substance present in the water phase, a limited effect of PVA concentration on NP-RBC adsorption will occur. However, if multiple species are present in the water phase, as is the case for PEI-PLGA NP synthesis, the effect of the surfactant becomes more complex. It can be expected that PEI and PVA will compete for adsorption onto the NPs, resulting in a mosaic of PEI-PVA on the NP surface. In follow-up experiments, it would be interesting to develop insights into the microstructure of the PEI-PLGA NP, to further clarify the precise effect of PVA concentration. However, from our results, it can be concluded that in order to maximize PLGA NP *ex vivo* adsorption efficiency and biocompatibility onto RBCs, it is advisable to keep PVA concentration to the minimum required in order to ensure the stability of the formulation.

#### Drug encapsulation effect

Multiple reports have illustrated the broad use of RBC hitchhiking for delivery of multiple drugs, such as doxorubicin and camptothecin [15, 22], however, the effect of these drugs in NP adsorption is not known. Understanding the effect of drug encapsulation on NP adsorption is crucial to capture the true versatility of the RBC hitchhiking platform. Therefore, we compared adsorption of PLGA, DEX-PLGA and PTX-PLGA NP onto human RBCs. Given the propensity of RBC-hitchhiked NPs to target the lungs, two drug formulations were selected that have shown successful incorporation into PLGA NPs [35, 36]. Both drugs are hydrophobic and thus suitable for nanoprecipitation synthesis, and have specific applications benefiting from targeted delivery to the lung. Paclitaxel (PTX) is widely used as a potent chemotherapeutic drug against non-small cell lung cancer [37], but suffers from poor solubility and thus limited bioavailability and off-target effects [38]. Dexamethasone (DEX) is a corticosteroid that has been widely explored for modulation of the inflammatory response to infectious diseases such as COVID-19 [39, 40]. Therefore, these two commonly used drugs were selected to demonstrate the effect of drug encapsulation on NP adsorption to RBCs.

**Fig. 5:**
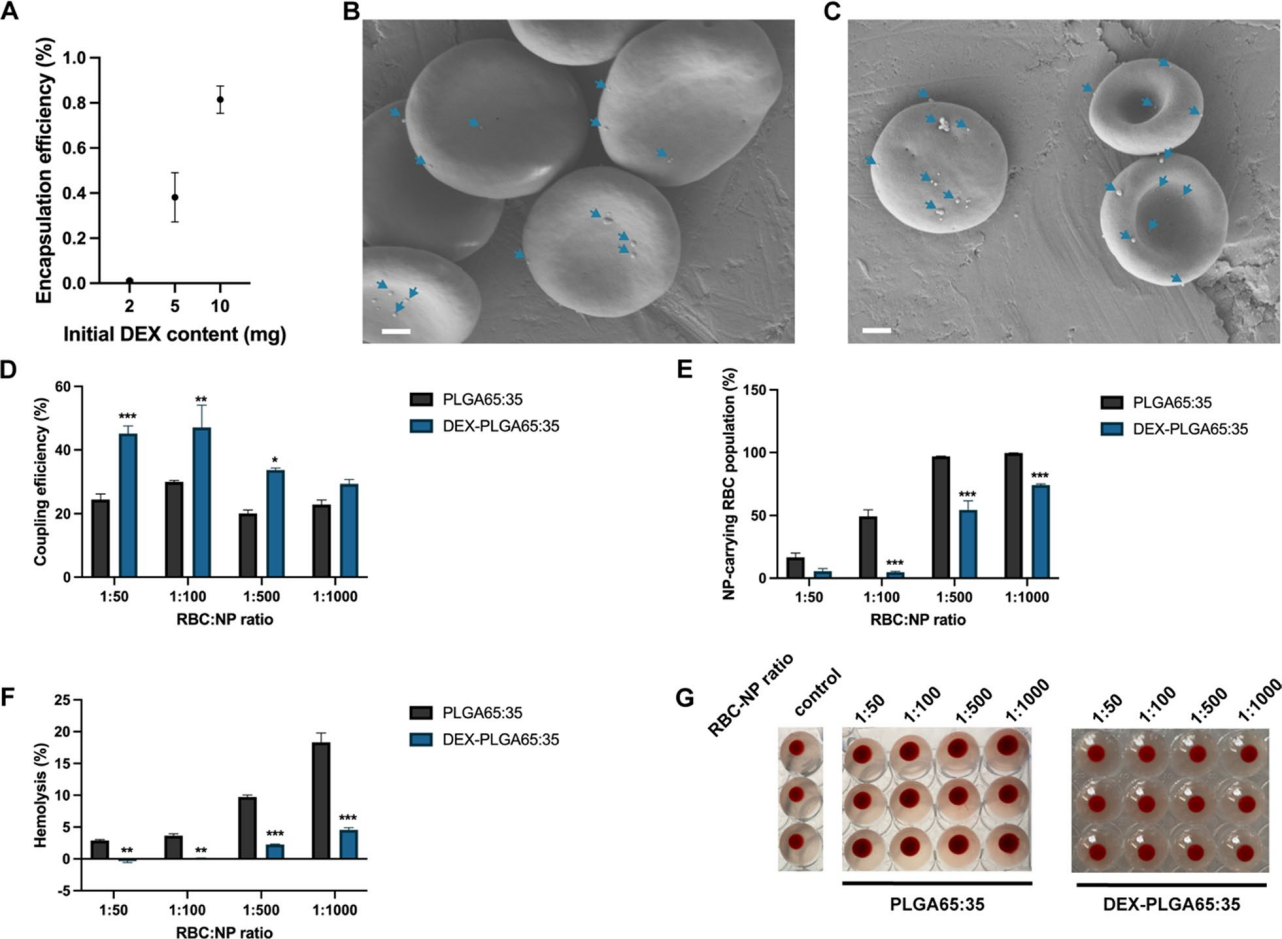
Effect of dexamethasone encapsulation on NP adsorption to human RBCs. **a** Evaluation of optimal initial dexamethasone content. 10 mg initial DEX content shows maximal and sufficient EE%. **b** Scanning electron microscopy images representing DEX-PLGA-NP adsorption onto human RBCs. **c** Scanning electron microscopy images representing DEX-PLGA-NP adsorption onto mouse RBCs. Scale bars indicate 1 µm. **d** Coupling efficiency of PLGA65:35 and DEX-PLGA65:35 NPs, onto human RBCs at different RBC:NP incubation ratios. **e** Percentages of human RBC populations carrying PLGA65:35 or DEX-PLGA65:35 NP. **f** Hemolysis percentages of human RBCs after incubation with PLGA65:35 or DEX-PLGA65:35 NPs at different RBC:NP incubation ratios. Hemolysis percentages are represented as total hemolysis subtracted by hemolysis of control cells. **g** Agglutination assay of human RBCs with PLGA65:35 or DEX-PLGA65:35 at different RBC:NP incubation ratios (n = 3). All data are presented as mean ± SEM (n = 3). The number of asterisks indicate the level of significance (two-way ANOVA with Sidak multiple testing adjustment), where: *p < 0.05, **p < 0.01 and ***p < 0.001

Our results showed that the encapsulation of DEX into PLGA65:35 NPs had only minor effects on size (212.57 ± 2.75 nm) and zeta potential (− 29.83 ± 0.60 mV) (Table 1 and Additional file 1: Fig. S15). Encapsulation of DEX achieved an encapsulation efficiency (EE%) of 75.79 ± 11.4 % at the optimal initial drug content of 10 mg (Fig. 5A). A drug release study of DEX-NPs shows an initial release burst in the first 6 h, followed by a sustained drug release over time (Additional file 1: Fig. S23), similarly to previously reported drug-loaded PLGA NP profiles [15]. After incubation of the DEX-NPs with RBCs, successful NP adsorption was verified by scanning electron microscopy (Fig. 5B,C), which revealed successful NP adsorption on RBCs. Additionally, NP adsorption results indicated, compared to plain PLGA-NPs, a 1.8-, 1.6- and 1.7-fold increase in coupling efficiency at incubation ratios of 1:50, 1:100 and 1:500 respectively (Fig. 5D), while a 10.2-, 1.8- and 1.3-fold decrease was detected in NP-carrying RBC population at incubation ratios of 1:100, 1:500 and 1:1000, respectively (Fig. 5E). Despite higher coupling efficiencies, a major reduction in hemolysis induction was observed, with, for example, a significant 5.7-fold reduction at the highest RBC:NP ratio (1:1000) (Fig. 5F), while no agglutination was detected at any RBC:NP ratio (Fig. 5G). These results suggest a stronger and more biocompatible affinity of DEX-PLGA NPs to adsorb onto RBCs compared to plain PLGA NPs, which can be attributed to the presence of DEX molecules present on the surface of the NPs. DEX has been shown to be tolerated in high concentrations by RBCs in previous reports [41, 42].

The encapsulation of PTX into PLGA65:35 NPs led to a slight increase in size (276.43 ± 9.06 nm) and decrease in zeta potential (27.93 ± 0.29) (Table 1 and Additional file 1: Fig. S16). Similarly to DEX NPs, adsorption results of PTX NPs to RBCs indicated, compared to plain PLGA NPs, a 2.0- and 1.6-fold increase in coupling efficiency at incubation ratios of 1:50 and 1:100, respectively (Additional file 1: Fig. S24A), while a major reduction in hemolysis induction was observed, with a 5.7-fold reduction at the highest 1:1000 ratio (Additional file 1: Fig. S24B). No agglutination was detected at any RBC:NP ratio (Additional file 1: Fig. S24D). Adsorption behavior of PTX-NPs did manifest differently as compared to DEX-NPs, with only at ratio 1:100 a higher NP-carrying RBC population compared to plain PLGA NP (Additional file 1: Fig. S24C).

Our results indicate that the presence of drug molecules on the NP surface strongly impacts NP adsorption outcomes by influencing non-covalent interactions. The drug encapsulation effect was shown here for DEX and PTX, of which the effect on NP physicochemical properties remained low. However, it can be expected that drugs that greatly influence the zeta potential of NPs, e.g. doxorubicin, will impact the NP coupling success and require thorough biocompatibility evaluation. Although our results confirmed the versatility of RBC hitchhiking for drug delivery purposes, the major influence of drug molecules on NP coupling calls for thorough NP adsorption investigation and optimization for every individual application of the RBC hitchhiking platform.

## Conclusions

In summary, this study demonstrates the effect of key parameters on NP-RBC adsorption, offering a framework of parameters to be accounted for. Such a framework is expected to aid researchers in rational selection of animal models and design of NPs. RBC origin was found to significantly impact NP adsorption outcome, a factor that should be attended to during selection of disease models. NP characteristics, such as hydrophobicity and zeta potential, were shown to require balancing between improving coupling efficiency and assuring biocompatibility. Additionally, despite the successful adsorption of different drug encapsulated NPs, the substantial effect of a specific drug on adsorption outcomes entails the need for meticulous characterization of each drug-NP-RBC complex and attenuates, together with effects of RBC and NP characteristics, RBC hitchhiking as a generic tool for drug delivery purposes. Thus, this study highlights the limits of ex vivo NP-RBC coupling with the current understanding and calls for more fundamental research on the nanoparticle-RBC interface.

## Supplementary Information

**Additional file 1: **Additional experimental details, method on dexamethasone encapsulation quantification, exemplary DLS graphs of nanoformulations, data on hydrophobicity and zeta potential effect on NP adsorption onto mouse and rabbit RBCs and data on optimal initial drug content.

## Data Availability

All data generated or analyzed during this study are included in this article.
