# Supplementary material for: Modularity of RBC hitchhiking with polymeric nanoparticles: testing the limits of non-covalent adsorption
Source: J Nanobiotechnology. 2022 Jul 16;20:333. doi: 10.1186/s12951-022-01544-0 (PMC9287723; doi:10.1186/s12951-022-01544-0)
Supplement: Supplementary file 1 — Additional file 1: Additional experimental details, method on dexamethasone encapsulation quantification, exemplary DLS graphs of nanoformulations, data on hydrophobicity and zeta potential effect on NP adsorption onto mouse and rabbit RBCs and data on optimal initial drug content. [file 12951_2022_1544_MOESM1_ESM.docx]

# Modularity of RBC hitchhiking with polymeric nanoparticles: testing the limits of non-covalent adsorption

*Vincent Lenders^a^, Remei Escudero^a^, Xanthippi Koutsoumpou^a^, Laura Armengol Álvarez^b^, Jef Rozenski^b^, Stefaan J. Soenen^a,c^, Zongmin Zhao^d,e^, Samir Mitragotri^f,g^, Pieter Baatsen^h,i^, Karel Allegaert^j,k,l,m^, Jaan Toelen^l,m,n^, Bella B Manshian^a,c*^*

1. Translational Cell and Tissue Research Unit, Department of Imaging and Pathology, KU Leuven, Herestraat 49, B3000 Leuven, Belgium
2. Medicinal Chemistry, Rega Institute for Medical Research, Department of Pharmaceutical and Pharmacological Sciences, KU Leuven, B3000 Leuven, Belgium
3. NanoHealth and Optical Imaging Group, Department of Imaging and Pathology, KU Leuven, Herestraat 49, B3000 Leuven, Belgium
4. Department of Pharmaceutical Sciences, College of Pharmacy, University of Illinois at Chicago, Chicago, IL 60612, USA
5. University of Illinois Cancer Center, Chicago, IL 60612, USA.
6. John A. Paulson School of Engineering and Applied Sciences, Harvard University, Cambridge, MA02138, USA
7. Wyss Institute of Biologically Inspired Engineering, Harvard University, Boston, MA02115, USA
8. VIB-KU Leuven Center for Brain and Disease Research Electron Microscopy Platform of the VIB Bioimaging Core, Leuven, Belgium
9. Department of Neurosciences, Leuven Brain Institute, KU Leuven, B3000 Leuven, Belgium
10. Department of Hospital Pharmacy, Erasmus MC University Medical Center, 3015, CN Rotterdam, the Netherlands
11. Clinical Pharmacology and Pharmacotherapy, Department of Pharmaceutical and Pharmacological Sciences, KU Leuven, B3000 Leuven, Belgium
12. Leuven Child and Youth Institute, KU Leuven, 3000 Leuven, Belgium
13. Woman and Child, Department of Development and Regeneration, KU Leuven, 3000 Leuven, Belgium
14. Department of Pediatrics, University Hospitals Leuven, 3000 Leuven, Belgium

Supplementary materials and methods

**Materials**

PLGA Resomer^®^ RG 503 H (acid terminated, lactide:glycolide 50:50, Mw 24,000-38,000), PLGA Resomer^®^ RG 653 H (acid terminated, lactide:glycolide 65:35, Mw 24,000-38,000), poly(vinyl alcohol) (Mw 31,000-50,000; 98-99% hydrolyzed), polyethylenimine (Branched, Mw 25,000 by LS) and Dexamethasone (USP reference standard) were purchased from Sigma-Aldrich (Belgium). Cyanine7.5 NHS ester was purchased from Lumiprobe (Germany). Paclitaxel was purchased from MedChemExpress (Belgium). Dulbecco’s Phosphate Buffered Saline (Gibco), Axygen™ 1.5-ml self-standing screw cap tubes, Greiner Bio-One™ Vacuette™ K2E K2 EDTA, Greiner Bio-One™ Vacuette™ Heparin and BD Vacutainer™ Citrate blood collection tubes were ordered from Fisher Scientific (Belgium).

**Flow cytometry analysis**

Flow cytometry data was analyzed using IDEAS 6.2 software. Analysis and gating strategies are illustrated in Supplementary Figure 1.

**Quantification of dexamethasone encapsulation**

After synthesis, the NPs were washed with deionized (DI) water (3x). In the final washing step, the pellet was not resuspended, but instead, dried for 4 hours in a desiccator to remove any hydrophilic supernatant. When the pellet was completely dry, it was resuspended in acetone and kept overnight under rotation. Depending on the expected incorporation, different solutions in methanol:water (1:1 v/v) were made, in order to confirm that the measured concentration would fall in the validated calibration curve. The chromatographic separation of analytes was successfully achieved on an Alliance 2965 Separation module (Waters, Milford, MA) HPLC system consisting of an autosampler, a built- in quaternary pump and 4-channel degasser. The chromatographic separation was carried out using a XBridge® C18 (2.1 x 50 mm, 3.5 μm particle size) column. The analysis was performed using a mobile phase consisting of a solvent mixture of water (eluent A) and ACN:water (1:1 v/v) (eluent B), in gradient elution mode. Linear gradient was from 10% B (0 min) to 100% B (10 min). Mobile phases in this study were prepared with the use of organic solvents for HPLC, including acetonitrile (HPLC grade, Chem-Lab NV®, Zedelgem, Belgium), and methanol (HPLC grade, Acros Organics®, Geel, Belgium). The system was pumped at a flow rate of 0.20 mL min -1 at room temperature, with a sample injection volume set at 2 μL for all analyses. Absorbance was measured at 240nm using a Waters 996 Photodiode Array (PDA). The total chromatographic run time was 17.5 minutes and the analytical system control, acquisition and data processing were performed using Mass Lynx software, version 4.1, from Waters Laboratory Informatics.

Optimal initial dexamethasone (DEX) content was determined by measuring the encapsulation efficiency of plain PLGA NPs synthesized with 2, 5 or 10 mg initial DEX content in the organic phase.

**DEX release quantification**

DEX loaded nanoparticles were resuspended in 1 mL PBS and incubated at 37° C on a tube revolver. At regular time points, the particles were centrifuged at 12.000 rpm for 10 mins. Samples were analyzed at 0, 1, 2, 3, 6, 12, 18, 24 and 48 hours after starting the incubation. The pellet was dried for 4 hours in a desiccator to remove any hydrophilic supernatant. When the pellet was completely dry, it was resuspended in acetone and kept overnight under rotation. The drug content in the pellet was quantified using HPLC, as described earlier, and was compared to the initial drug content to calculate the release percentage.

**DEX encapsulation quantification method validation**

The LC-UV method for DEX EE% quantification was validated using a single-laboratory approach to method validation, according to the appropriate guidelines described by the European Medicines Agency (EMEA), the Eurachem guide, the Food and Drug Administration (FDA) guidance for industry, and the International Council for Harmonisation of Technical Requirements for Pharmaceuticals for Human Use (ICH). The method was validated on its linearity, precision, accuracy, peak symmetry, limit of detection (LOD), limit of quantification (LOQ), matrix effect and selectivity.

***Determination of maximum absorption***

By the appropriate dilution of pure DEX with methanol water (1:1 v/v), a solution was made and scanned in the range of 210-400nm to determine the wavelength of maximum absorption. A UV spectrophotometer (UV-1800 Shimadzu Corporation®), was used to select the optimal UV wavelength (lmax) for the detection of DEX in the standard solution as well as in PLGA NPs. The analyses were carried out using methanol water (1:1 v/v) as blank. The wavelength of maximum absorbance (lmax) of the drug was 240nm, as shown in Supplementary Figure 2.

***Statistical validation parameters***

*Linearity*

To establish linearity, seven calibration standards were prepared within the concentration range of 10 to 100 μg mL-1 and were run in duplicate. The six concentration levels used (10, 20, 40, 60, 80, 100 μg mL-1) were analyzed in two independent runs. In Supplementary Table 1, the preparation of the calibration standards is shown.

*Lower Limit of Quantification*

The lowest standard on the calibration curve was defined as the LLOQ. For its determination, six consecutive runs of one sample of the lowest concentration (20 μg mL-1) were analyzed consecutively and the RSD (%) of the results was calculated.

*Precision*

The analytical precision of the study was determined by assessing the repeatability (intra-day) and intermediate precision (inter-day) and was reported as RSD%. Two different concentrations were analyzed: 20 and 100 μg mL-1. Repeatability was evaluated by measuring each concentration six times on the same day (n=6/day), during three consecutive days. Intermediate precision was determined by analyzing all the results for the three days (n=18). Significance was evaluated through one-way ANOVA with a 95% confidence level.

*Accuracy*

Accuracy of the study was established across the concentration range of 10 to 100 μg mL-1. Three concentration levels, covering the specified range, were measured (10, 40 and 100 μg mL-1) and 3 different solutions were prepared for each concentration level.

*Peak symmetry*

Symmetry factor was determined with tailing factor equation:

$T=\frac{W_{0.05h}}{2f} \mathrm{with}W0.05 the peak at 5\% height and f the width \left( \mathrm{time} \right)$ $between the peak maximum and the front edge of the peak at 5\% of the peak height.$

Number of theoretical plates (N) was determined using:

$N=5.54x\left( \frac{t_{r}}{W_{0.5b}} \right)^{2} with t_{r} retention time and W_{0.5b}$peak width at 5% of the peak height

*Sensitivity*

LOD and LOQ were calculated through the results obtained from the linearity calibration curve, using the same concentration levels with the same two replicates for each level.

*Matrix effect*

The matrix effect was evaluated by comparing the peak areas of PLGA NPs samples spiked with the calibration standard solution of DEX (0.1 mg mL-1) with the peak areas of the same standard solution. The two groups in comparison (Supplementary Table 2) were prepared in triplicate.

*Selectivity (or specificity)*

Selectivity was determined by running a blank on a sample without analyte (DEX).

**Supplementary results and discussion**

**DEX encapsulation quantification**

*Linearity*

The integrated peak areas were calculated and integration results from the two independent runs are available in Supplementary Table 3. The calibration curve was constructed by linear regression analysis (Supplementary Figure 3). The equation for the representative linear calibration curve determined was 𝑦 = 4087.40𝑥 − 3267.93, with y and x the integrated areas and DEX concentrations, respectively. The coefficient of determination r^2^ was 0.9994, with a 95% confidence interval for the slope and the intercept. In Supplementary Table 4, statistical results for the calibration curve are given. The assay shows linearity over the concentration range from 10 to 100 μg mL-1, meaning that the linear regression model was acceptable for the selected analyte in the defined range with a satisfactory coefficient of determination (r^2^) above 0.99, indicating a high correlation between the tested variables.

*Lower Limit of Quantification*

The results of the six analytical runs of the lowest standard of calibration are displayed in Supplementary Table 5. Further calculations of the mean and standard deviation (SD) show an RSD% value of 1.50% (Supplementary Table 6). The RSD% for the lowest concentration of the calibration curve was 1.50%, which is lower than the requirement of the validation guidelines (< 20%).

*Precision*

Supplementary Tables 7 and 8 show repeatability and intermediate precision results for concentrations 20 and 100 μg mL-1, respectively. The RSD% values for repeatability and intermediate precision do not exceed 15% for both samples. Subsequently, the precision on the peak area of the assays is within the acceptance criteria of the reference guidelines. Q-Q plots (Supplementary Figures 4 and 5) and the result of the Shapiro Test (*p > 0.05*) determined that the data was normally distributed. In addition, residual plots (Figures 15 and 17), the boxplot (Supplementary Figures 6 and 7) and the Levene’s Test result (*p > 0.05*) demonstrated homogeneity, so that a one-way ANOVA could be performed. The p-value of ANOVA indicated the existence of significant differences between the groups. Performing a Tukey test, it was found that for the lowest concentration, there are significant differences between day 3 and 2, while for the highest concentration, there are significant differences between day 3 and 1 and between day 3 and 2. Results of all the performed tests are displayed in Supplementary Table 9.

*Accuracy*

Accuracy is reported as the difference between the mean and true value with confidence intervals. The statistical analysis of accuracy is presented in Supplementary Table 10. The minimal differences between the calculated concentrations and the expected values (RSD < 15%) indicated that the accuracy of the tested analytical method was good.

*Peak symmetry*

Results of tailing and N showed an RSD < 15% and tailing factor close to unity (< 2), demonstrating good peak symmetry for the analyte (DEX) in determination (Supplementary Table 11).

*Sensitivity*

After the establishment of linearity and through the linearity calibration curve-, the LOD was found as 1.72 μg mL-1, while the LOQ was 5.67 μg mL-1. In Supplementary Table 12, the results used for the determination of both limits are shown, considering Sa as the SD of the intercept.

*Matrix effect*

Comparisons between the groups of DEX and DEX-PLGA NPs were performed using independent samples t-test (Supplementary Table 13). No significant differences were found (p = 0.1564). Thus, no interference of the matrix was observed during the analysis of the analyte. The proposed method is specific for unequivocal determination of DEX in the presence of PLGA NPs.

*Selectivity (or specificity)*

Blanks of the matrix were analyzed, and the responses of the interfering peaks in the retention time of the analyte (DEX) were recorded. After running the blank, it was confirmed that there were no interfering peaks in the chromatograms of PLGA NPs, where DEX normally elutes. The peak at 1.6 min was due to the presence of acetone in the stock solution. In addition, for chromatograms from LLOQ samples of DEX, it was stated that each peak had good shape and was clearly separable from the other peaks. Selectivity is proven by showing the adequate chromatograms, displayed in Supplementary Figures 8 and 9.

**Supplementary Tables**

**Table S1.** Preparation of DEX calibration standards for linearity determination

**
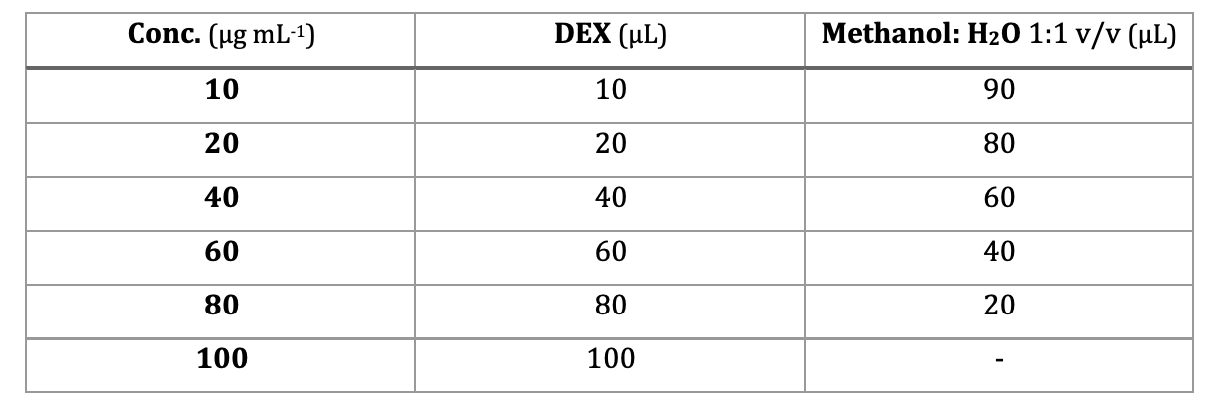
**

**Table S2.** Comparison groups for matrix effect testing


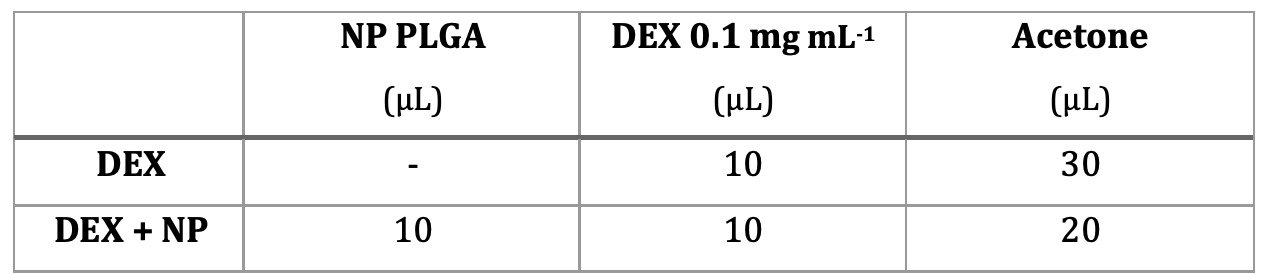


**Table S3.** Integrated areas of the 6 concentration levels for linearity determination


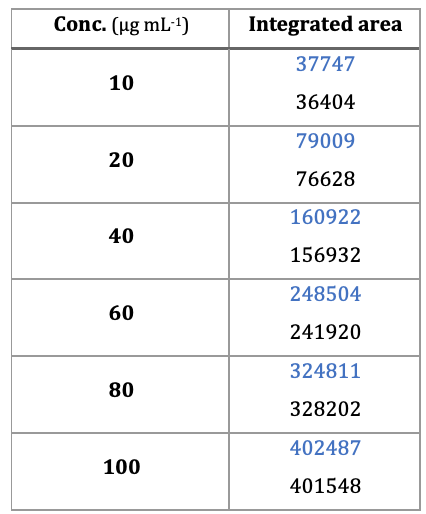


**Table S4.** Statistical results for the calibration curve after linear regression


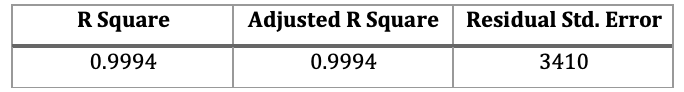


**Table S5.** Integrated areas of the lowest point of calibration curve


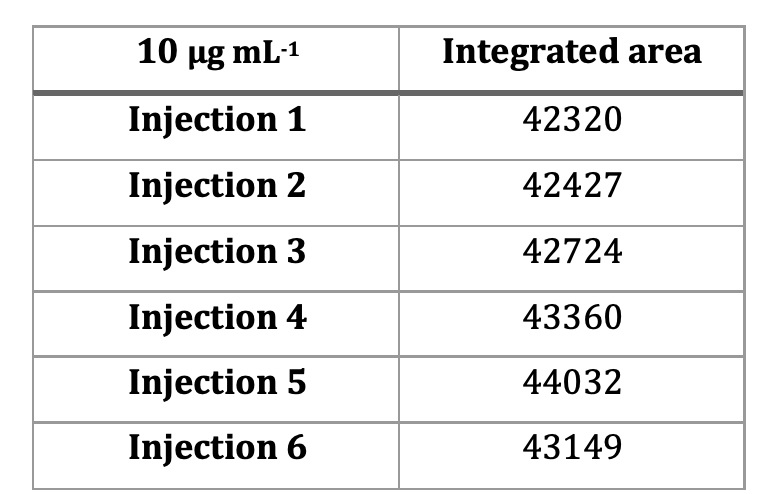


**Table S6.** Lowest point of calibration curve result expressed in RSD%


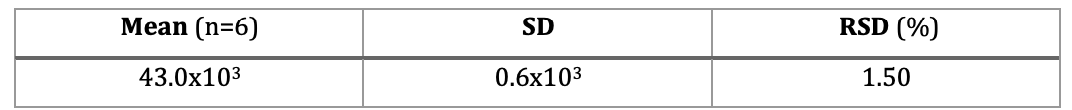


**Table S7.** Repeatability and intermediate precision results of the method for theorethical concentration 20 μg mL^-1^


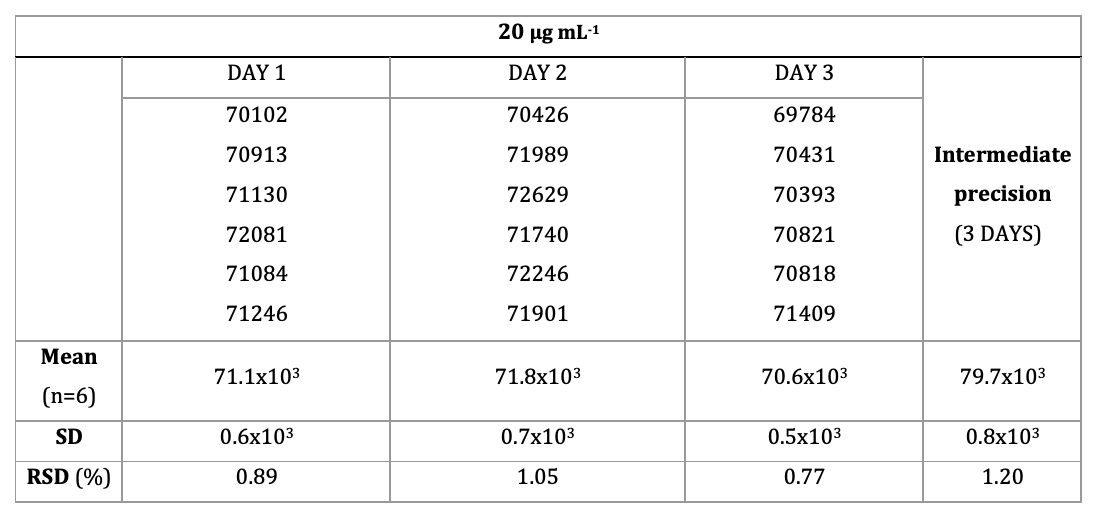


**Table S8.** Repeatability and intermediate precision results of the method for theorethical concentration 100 μg mL^-1^


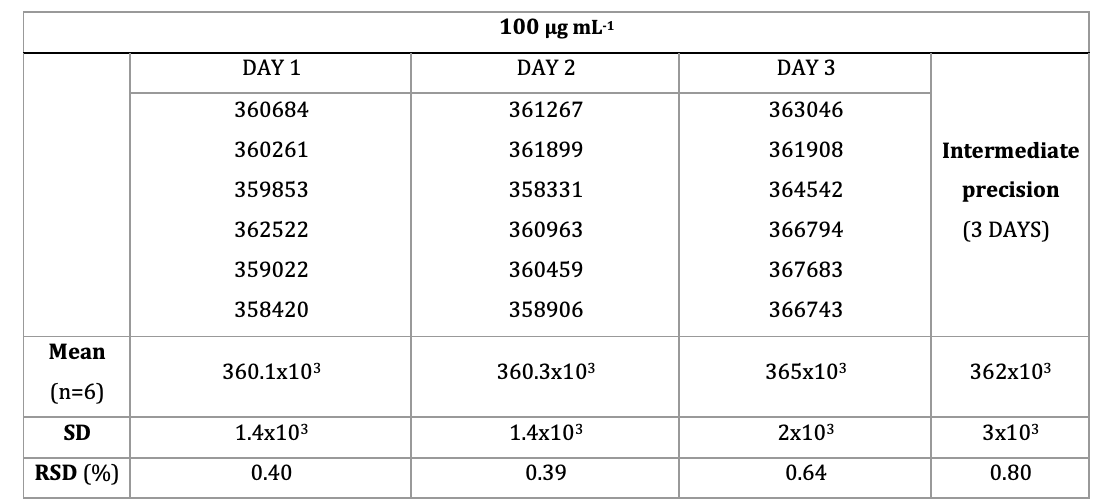


**Table S9.** Results for ANOVA assumptions and test of precision data


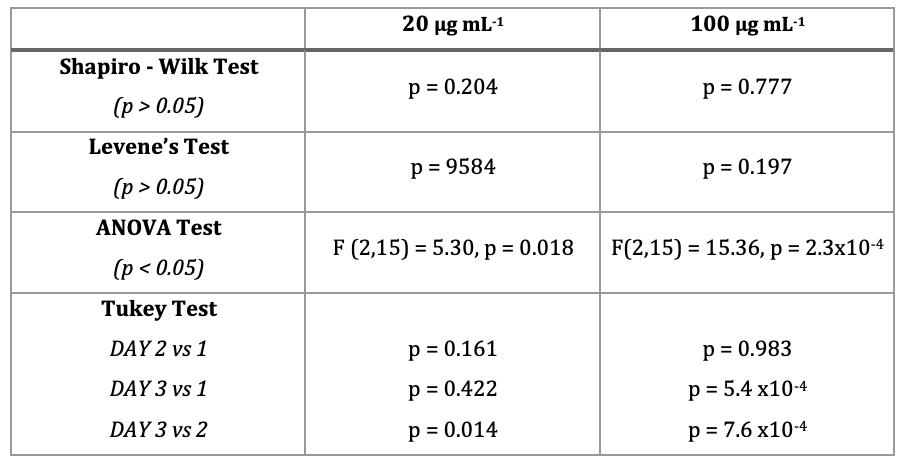


**Table S10.** Accuracy results for DEX for the theoretical concentrations of 10, 40 and 100 μg mL^-1^)

**
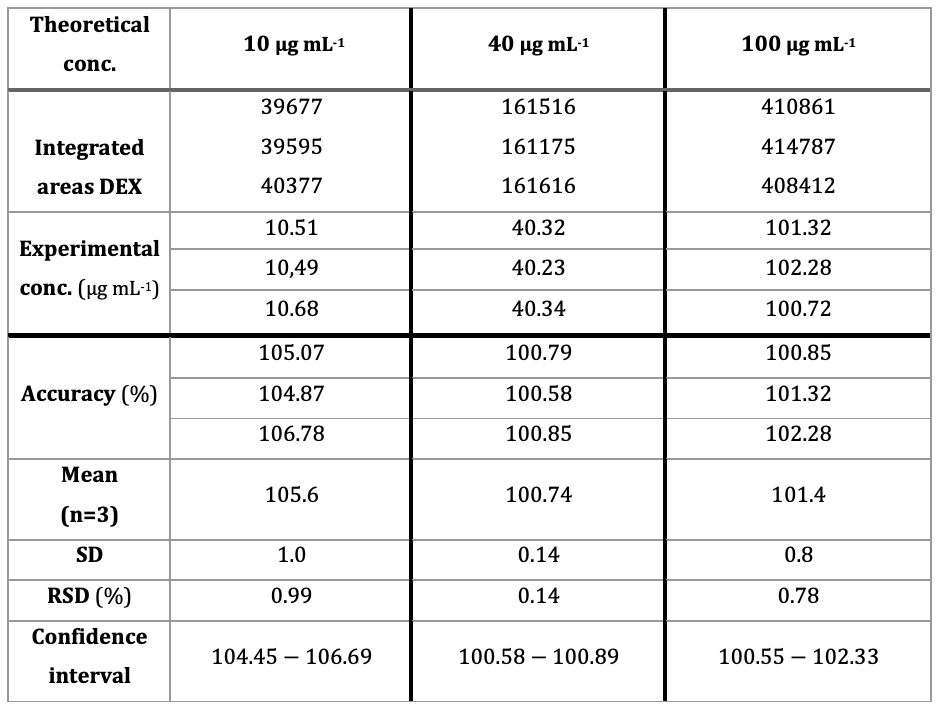
**

**Table S11.** Tailing factor and number of theoretical plates (N) for DEX

**
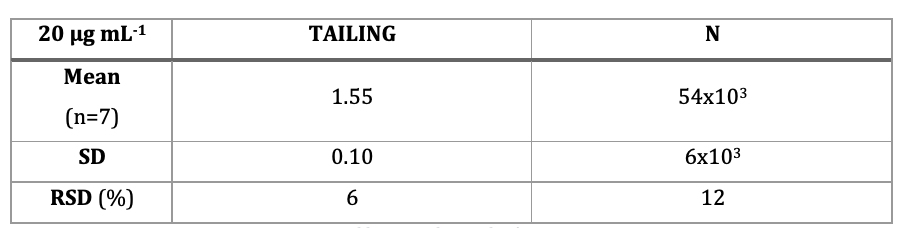
**

**Table S12.** Results for the determination of LOD and LOQ

**
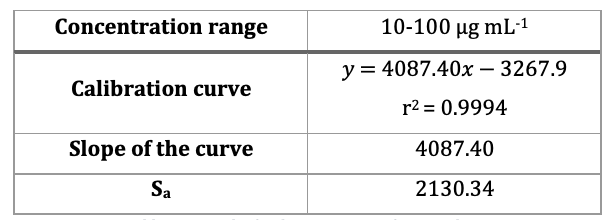
**

**Table S13.** Results for the matrix effect of DEX in PLGA nanoparticles

**
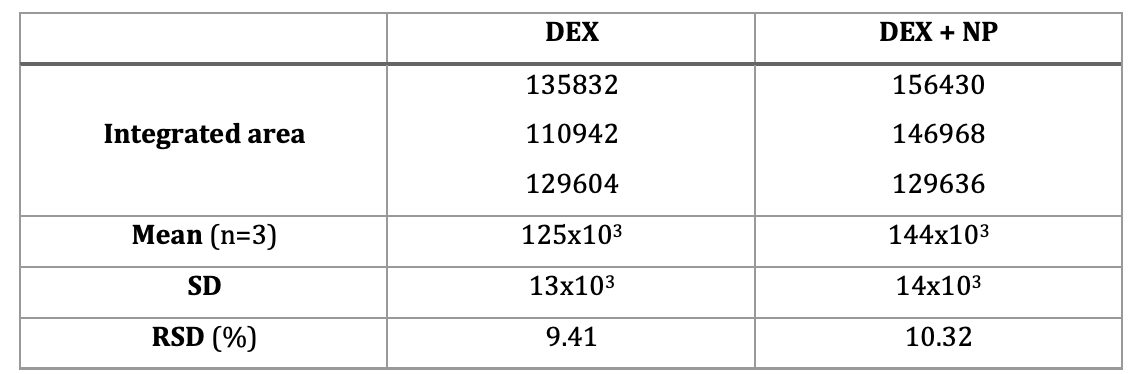
**

**Supplementary Figures**


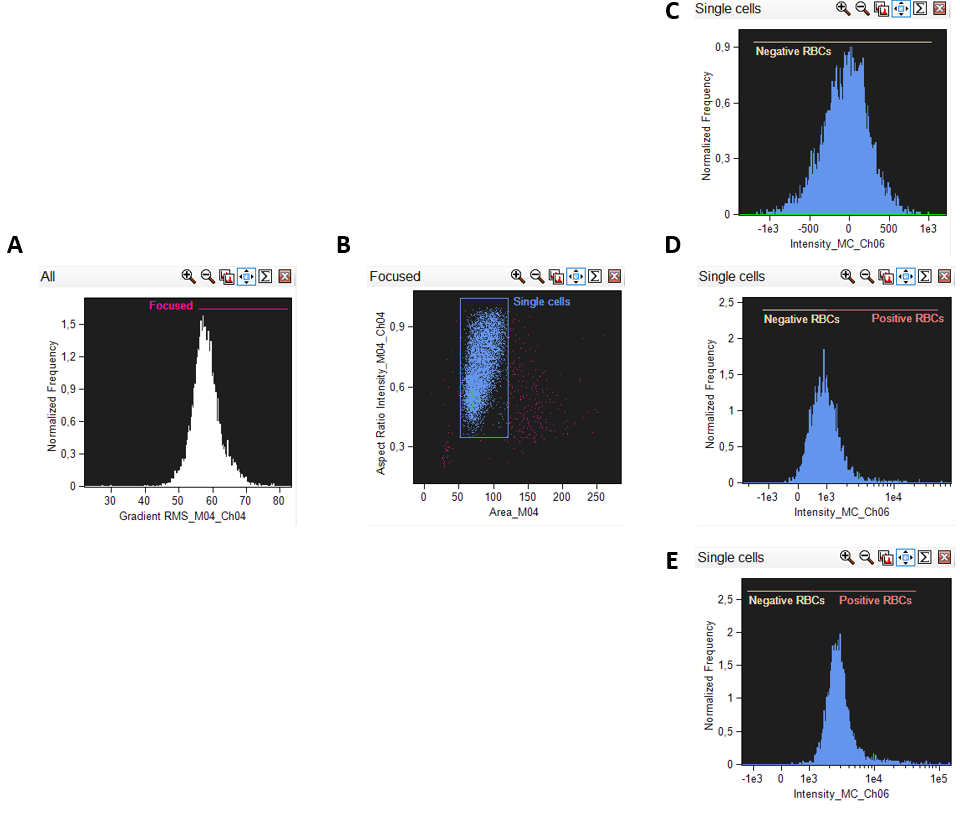


**Fig. S1.** Gating strategy for flow cytometry analysis. (**a**) From all imaged cells, focused cells were selected. (**b**) Single cells were then selected from the focused cell population. (**c**) Fluorescence intensity of control cells was selected as negative cells. Example shown for control human RBCs. (**d**) Fluoresence signal above control level was selected as positive cells. Example shown for human RBCs at an incubation ratio of 1:100 RBC:NPs . (**e**) Example shown for human RBCs at an incubation ratio of 1:1000.


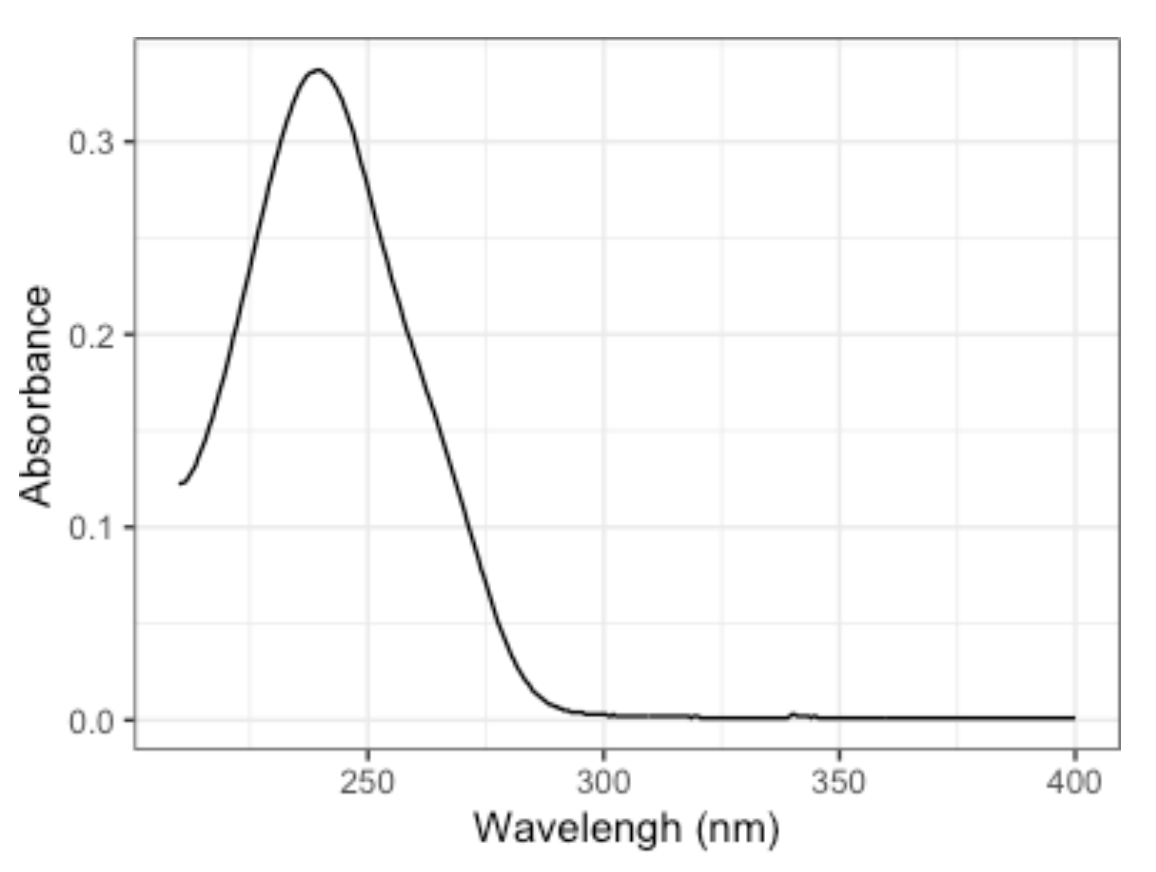


**Fig. S2** Graph representing the DEX absorbance value at 240nm.


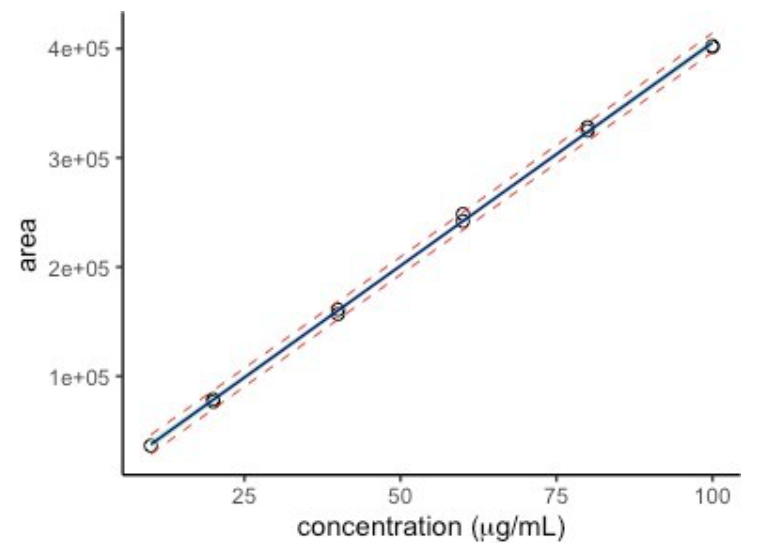


**Fig. S3** Linearity graph from standard solutions of DEX.


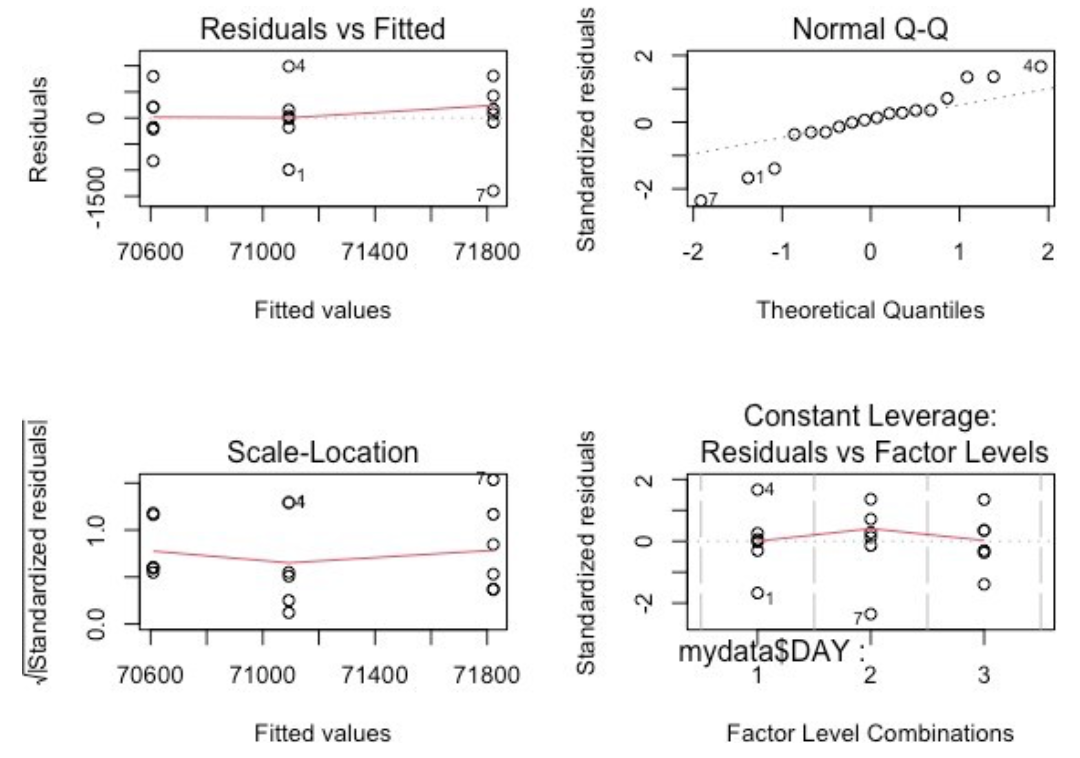


**Fig. S4** Precision plots for the concentration of 20 μg mL^-1^.


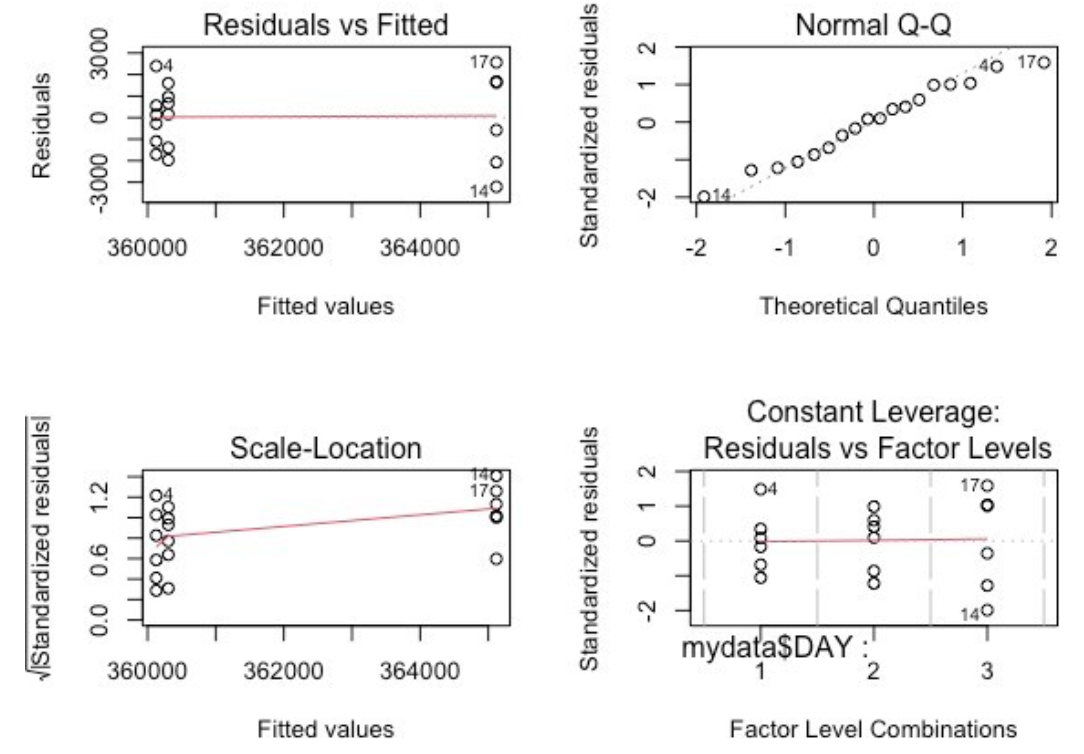


**Fig. S5** Precision plots for the concentration of 100 μg mL^-1^.

**
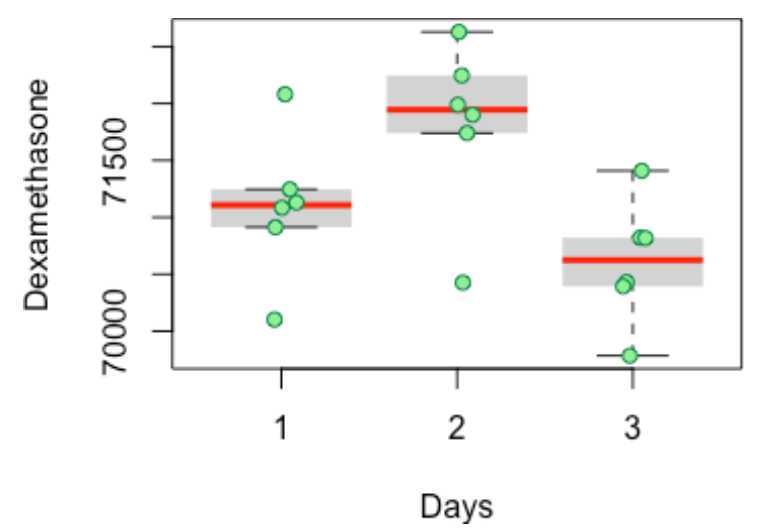
**

**Fig. S6** Boxplot for the concentration of 20 μg mL^-1^.

**
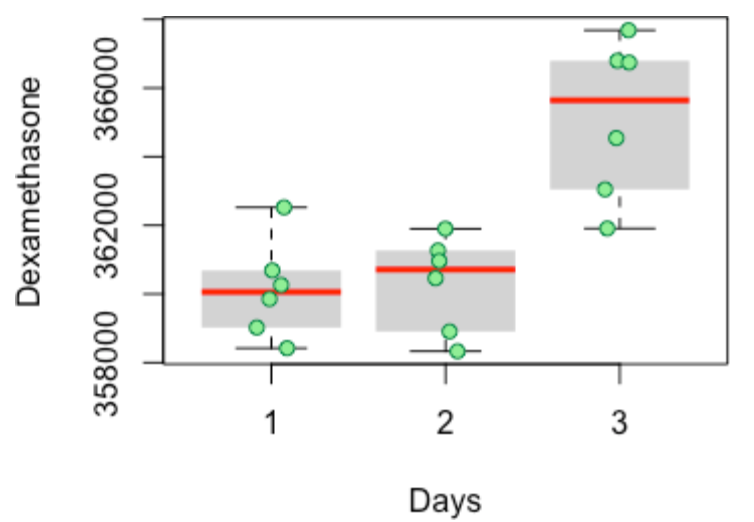
**

**Fig. S5** Boxplot for the concentration of 100 μg mL^-1^.


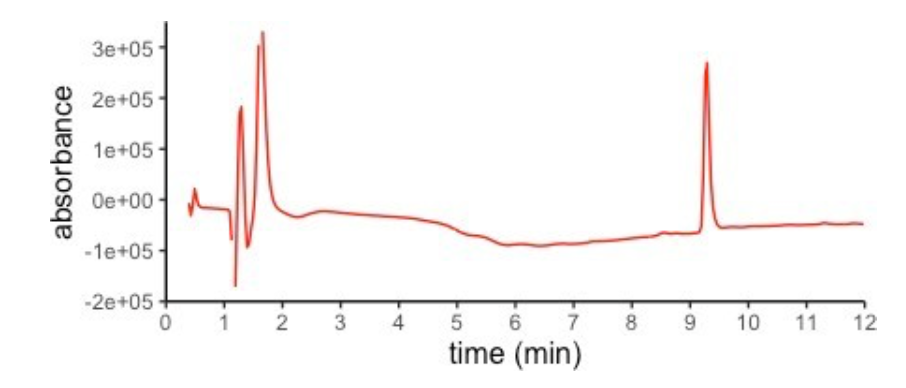


**Fig. S8** Representative chromatogram of LLOQ of DEX.


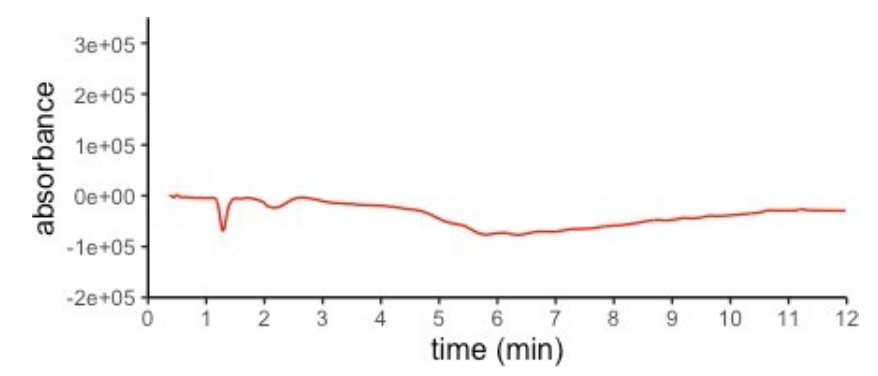


**Fig. S9** Representative chromatogram of a blank for DEX.


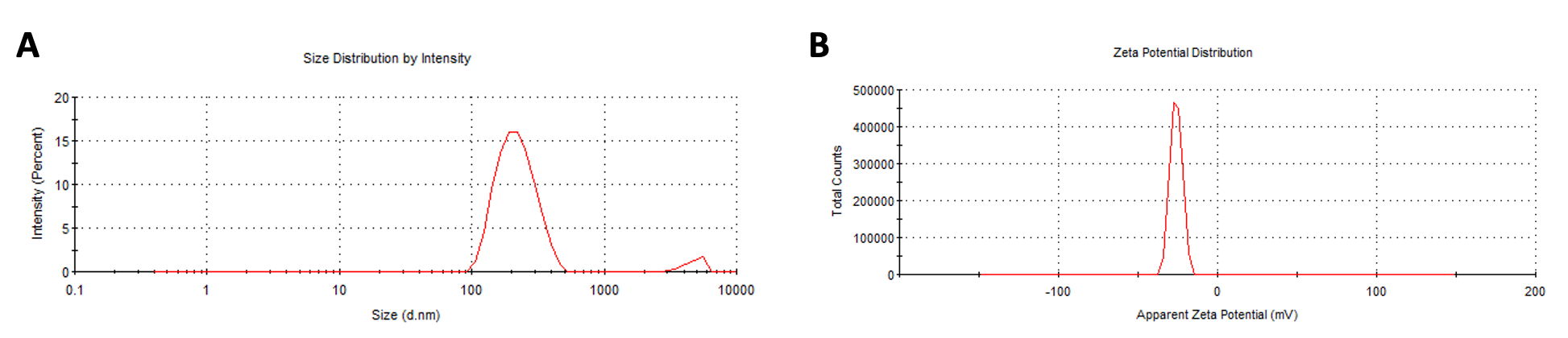


**Fig. S10** Exemplary DLS graphs for PLGA65:35 NPs with (**a**) size distribution and (**b**) Zeta potential distribution.


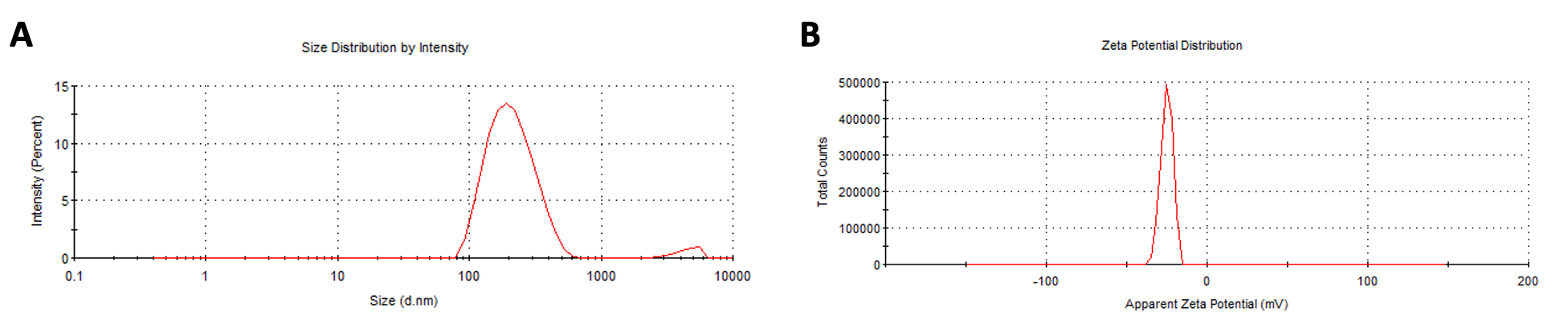


**Fig. S11** Exemplary DLS graphs for PLGA50:50 NPs with (**a**) size distribution and (**b**) Zeta potential distribution.


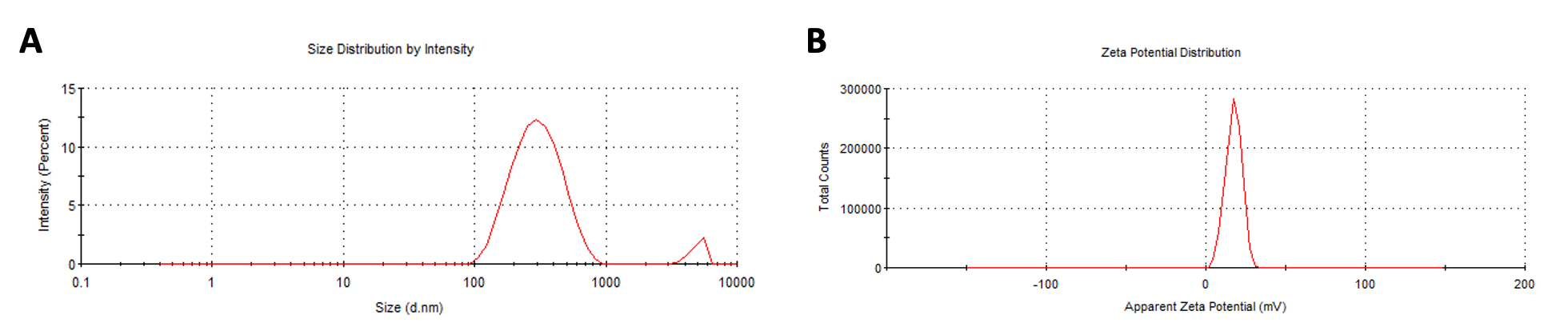


**Fig. S12** Exemplary DLS graphs for PEI-PLGA65:35 NPs with (**a**) size distribution and (**b**) Zeta potential distribution.


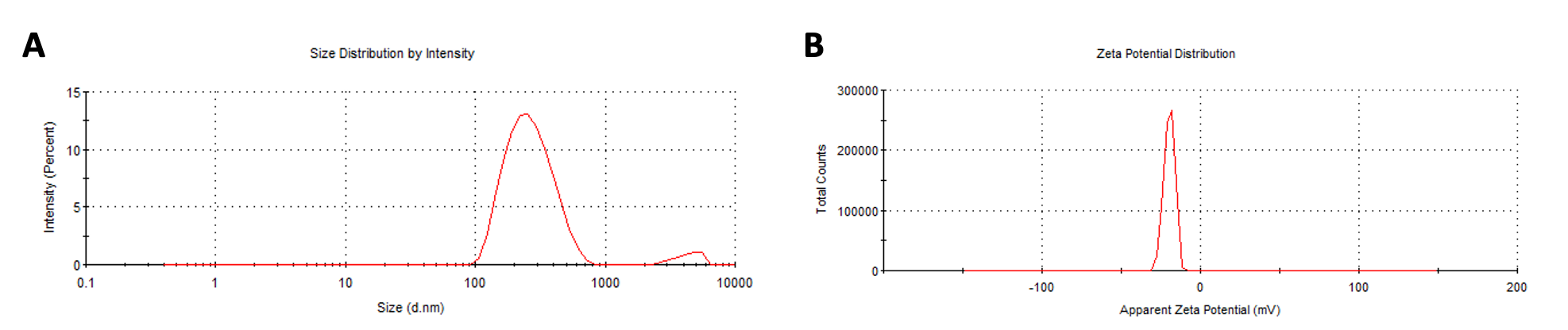


**Fig. S13** Exemplary DLS graphs for PLGA65:35 3%PVA NPs with (**a**) size distribution and (**b**) Zeta potential distribution.


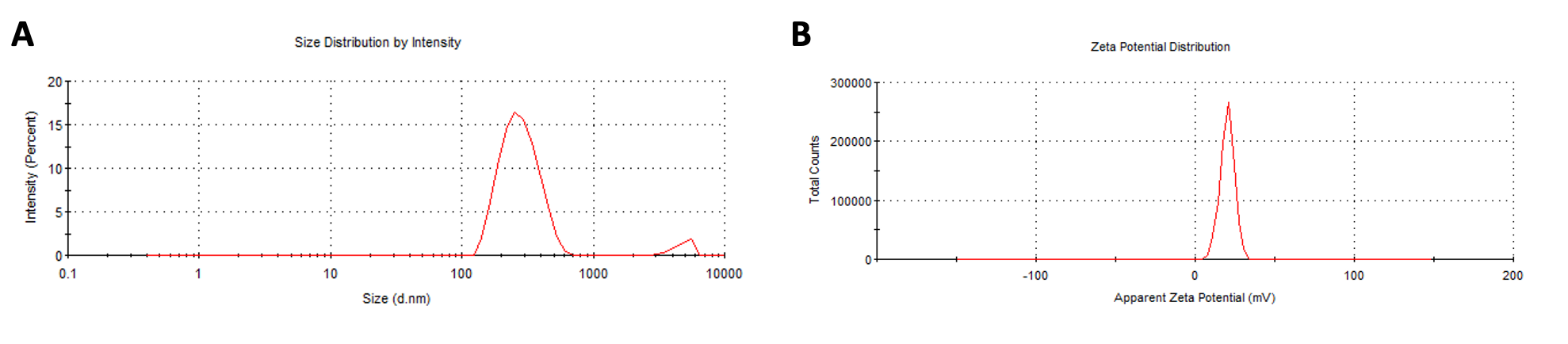


**Fig. S14** Exemplary DLS graphs for PEI-PLGA65:35 3%PVA NPs with (**a**) size distribution and (**b**) Zeta potential distribution.


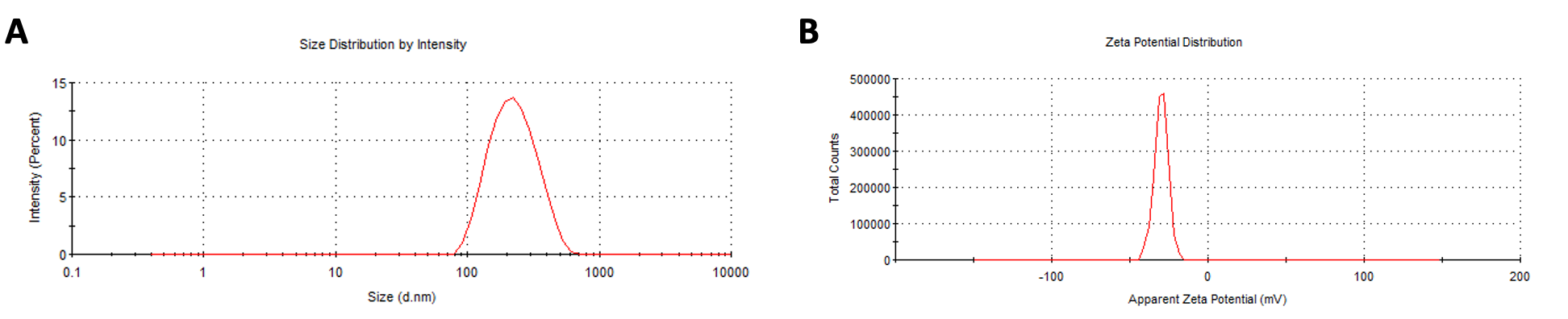


**Fig. S15** Exemplary DLS graphs for DEX-PLGA65:35 NPs with (**a**) size distribution and (**b**) Zeta potential distribution


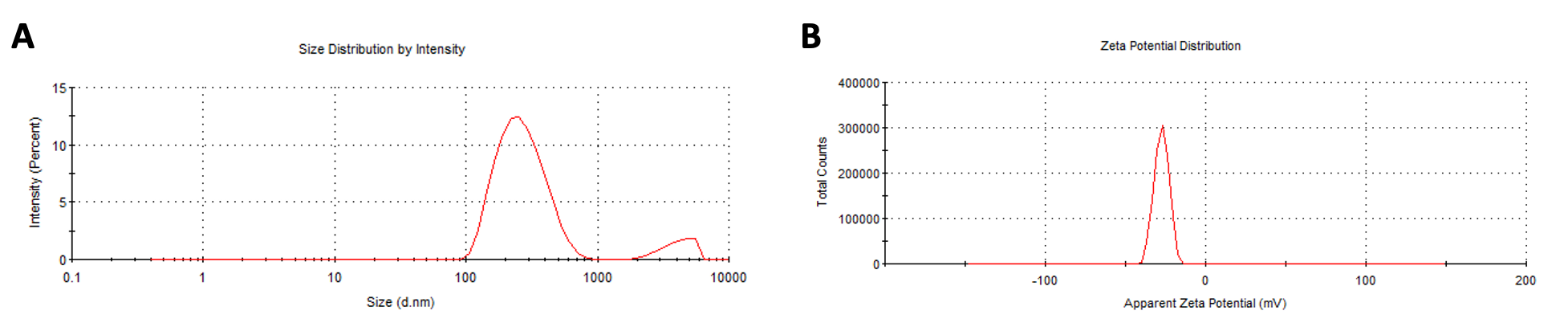


**Fig. S16** Exemplary DLS graphs for PTX-PLGA65:35 NPs with (**a**) size distribution and (**b**) Zeta potential distribution.


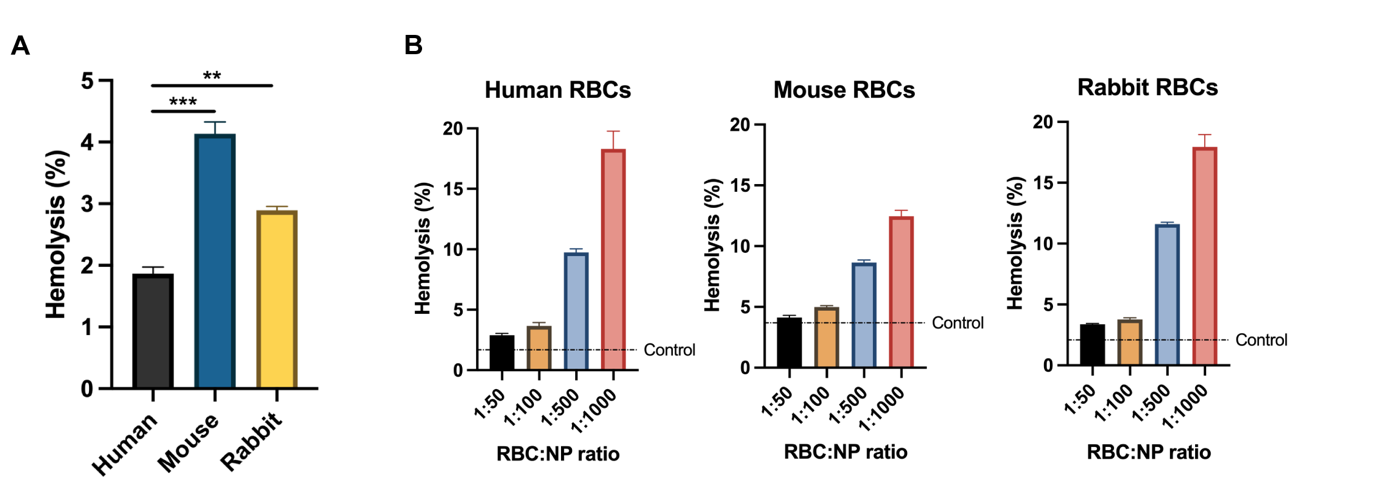


**Fig. S17** Hemolysis induction due to PLGA NP adsorption onto human, mouse and rabbit RBCs. (**a**) Hemoysis percentages of control RBCs. The number of asterisks indicate the level of significance (one-way ANOVA with Dunnet multiple testing adjustment): **P<0.01 and ***P<0.001. (**b**) Hemolysis percentages of different RBC types at different RBC:NP incubation ratios. Hemolysis percentages of respective control cells are indicated on the graphs. All data are presented as mean ± SEM (n = 3).

**Fig. S18** Effect of different anti-coagulants on the coupling efficiency of PLGA NP on human erythrocytes. Shown for incubation RBC:NP ratio 1:1000. All data are presented as mean ± SEM (n = 3). The number of asterisks indicate the level of significance (one-way ANOVA with Tukey multiple testing adjustment): **P<0.01 and ***P<0.001.


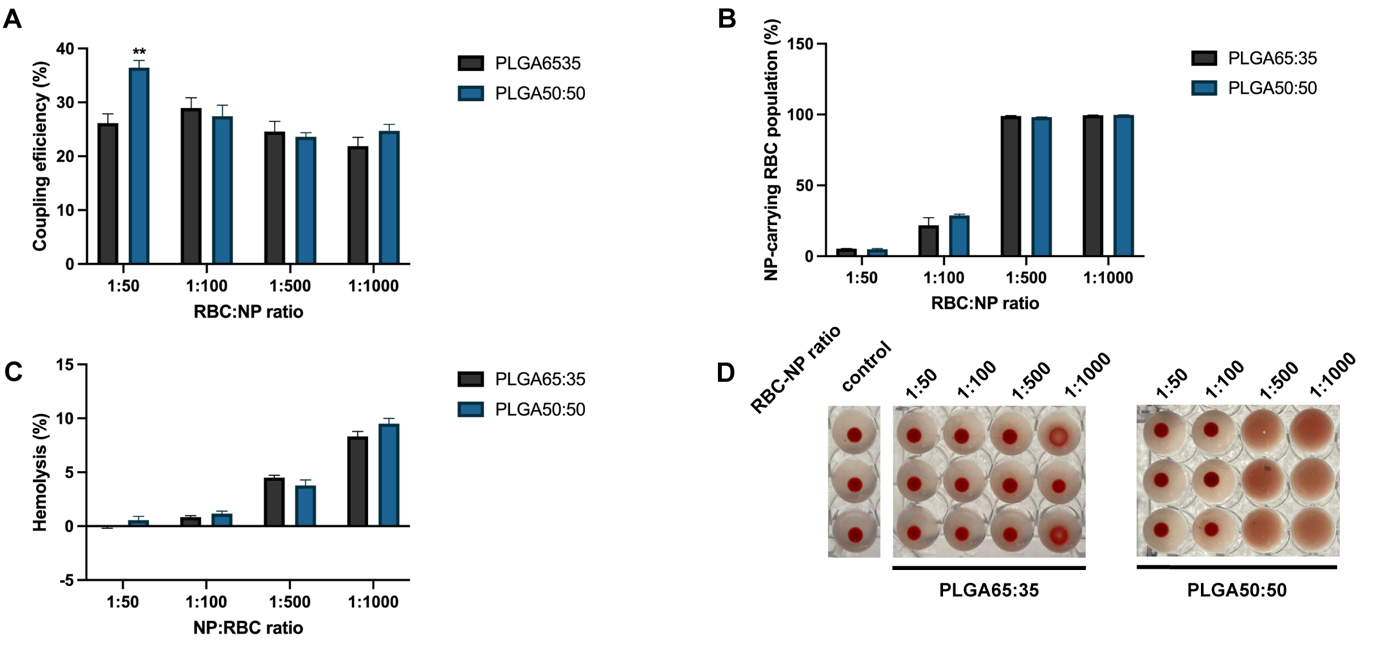


**Fig. S19** Hydrophobicity effect on NP adsorption onto mouse RBCs. (**a**) Coupling efficiency of PLGA65:35 and PLGA50:50 nanoparticles onto mouse RBCs at different RBC:NP incubation ratios. (**b**) Percentages of mouse RBC populations carrying PLGA65:35 or PLGA50:50 NP. (**c**) Hemolysis percentages of mouse RBCs after incubation with PLGA65:35 or PLGA50:50 NPs at different RBC:NP incubation ratios. Hemolysis percentages are represented as total hemolysis subtracted by hemolysis of control cells. (**d**) Agglutination assay of mouse RBCs with PLGA65:35 or PLGA50:50 NPs at different RBC:NP incubation ratios (n=3). All data are presented as mean ± SEM (n = 3). The number of asterisks indicate the level of significance (two-way ANOVA with Sidak multiple testing adjustment), where: ** = p < 0.01.


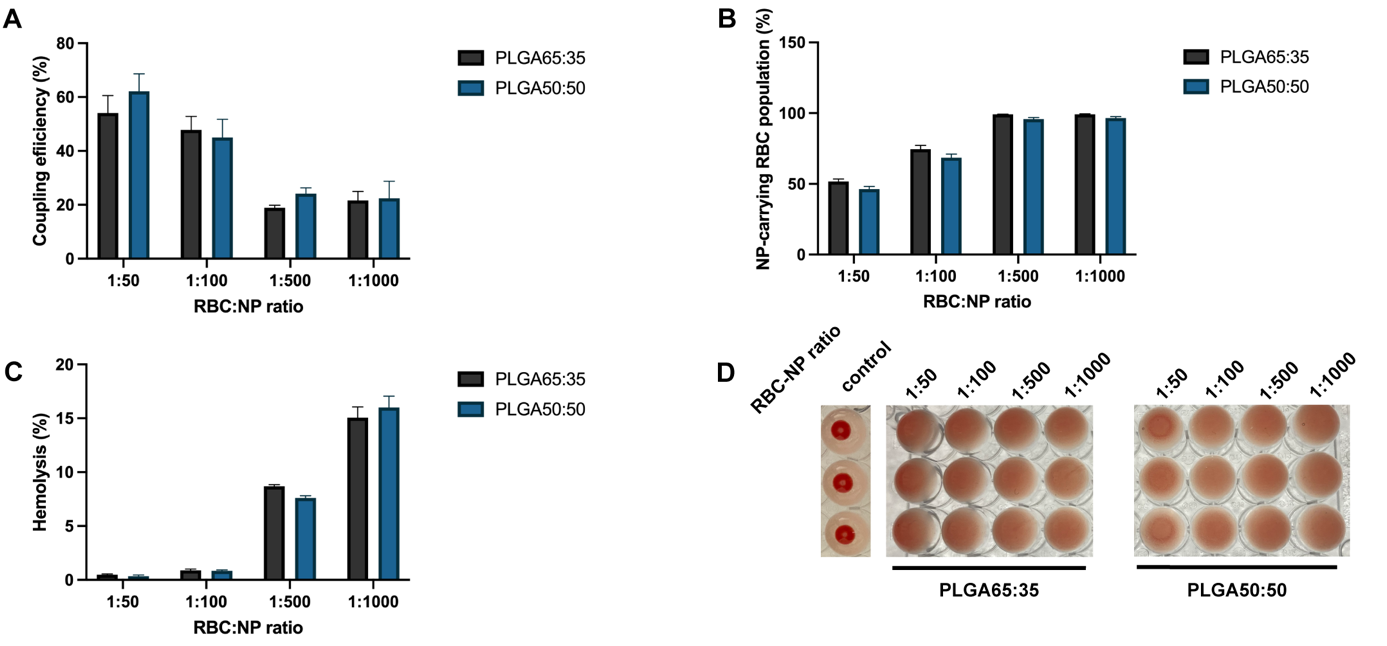


**Fig. S20** Hydrophobicity effect on NP adsorption onto rabbit RBCs. (**a**) Coupling efficiency of PLGA65:35 and PLGA50:50 nanoparticles onto rabbit RBCs at different RBC:NP incubation ratios. **(b**) Percentages of rabbit RBC populations carrying PLGA65:35 or PLGA50:50 NP. (**c**) Hemolysis percentages of rabbit RBCs after incubation with PLGA65:35 or PLGA50:50 NPs at different RBC:NP incubation ratios. Hemolysis percentages are represented as total hemolysis subtracted by hemolysis of control cells. (**d**) Agglutination assay of rabbit RBCs with PLGA65:35 or PLGA50:50 NPs at different RBC:NP incubation ratios (n=3). All data are presented as mean ± SEM (n = 3).


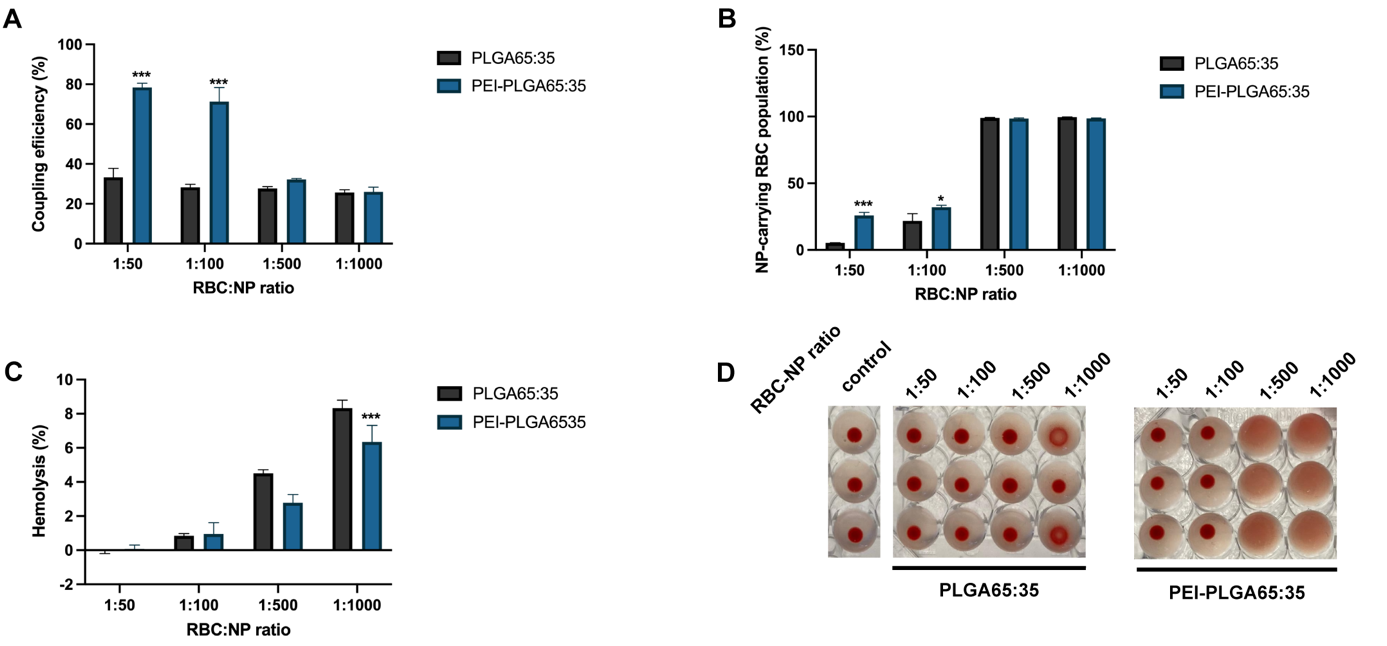


**Fig. S21** Zeta potential effect on NP adsorption onto mouse RBCs. (**a**) Coupling efficiency of PLGA65:35 and PEI-PLGA65:35 nanoparticles onto mouse RBCs at different RBC:NP incubation ratios. (**b**) Percentages of mouse RBC populations carrying PLGA65:35 or PEI-PLGA65:35 NP. (**c**) Lysis percentages of mouse RBCs after incubation with PLGA65:35 or PEI-PLGA65:35 NPs at different RBC:NP incubation ratios. Hemolysis percentages are represented as total hemolysis subtracted by hemolysis of control cells. (**d**) Agglutination assay of mouse RBCs with PLGA65:35 or PEI-PLGA65:35 NPs at different RBC:NP incubation ratios (n=3). All data are presented as mean ± SEM (n = 3). The number of asterisks indicate the level of significance (two-way ANOVA with Sidak multiple testing adjustment), where: * = p < 0.05 and *** = p < 0.001.


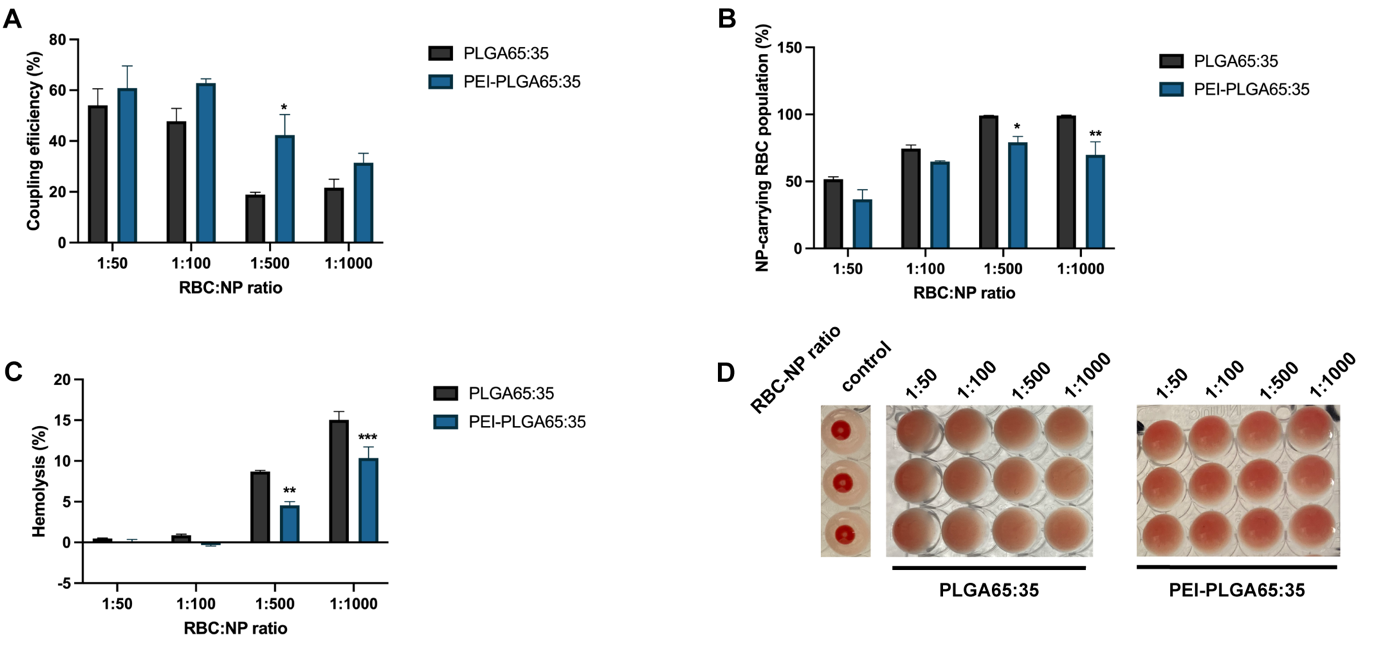


**Fig. S22** Zetapotential effect on NP adsorption onto rabbit RBCs. (**a**) Coupling efficiency of PLGA65:35 and PEI-PLGA65:35 nanoparticles onto rabbit RBCs at different RBC:NP incubation ratios. (**b**) Percentages of rabbit RBC populations carrying PLGA65:35 or PEI-PLGA65:35 NP. (**c**) Hemolysis percentages of rabbit RBCs after incubation with PLGA65:35 or PEI-PLGA65:35 NPs at different RBC:NP incubation ratios. Hemolysis percentages are represented as total hemolysis subtracted by lysis of control cells. (**d**) Agglutination assay of rabbit RBCs with PLGA65:35 or PEI-PLGA65:35 NPs at different RBC:NP incubation ratios (n=3). All data are presented as mean ± SEM (n = 3). The number of asterisks indicate the level of significance (two-way ANOVA with Sidak multiple testing adjustment), where: * = p < 0.05, ** = p < 0.01 and *** = p < 0.001.


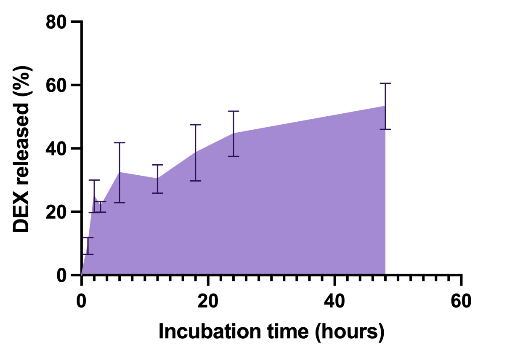


**Fig. S23** Drug release profile of DEX-loaded NPs in PBS. All data are presented as mean ± SEM (n = 3).


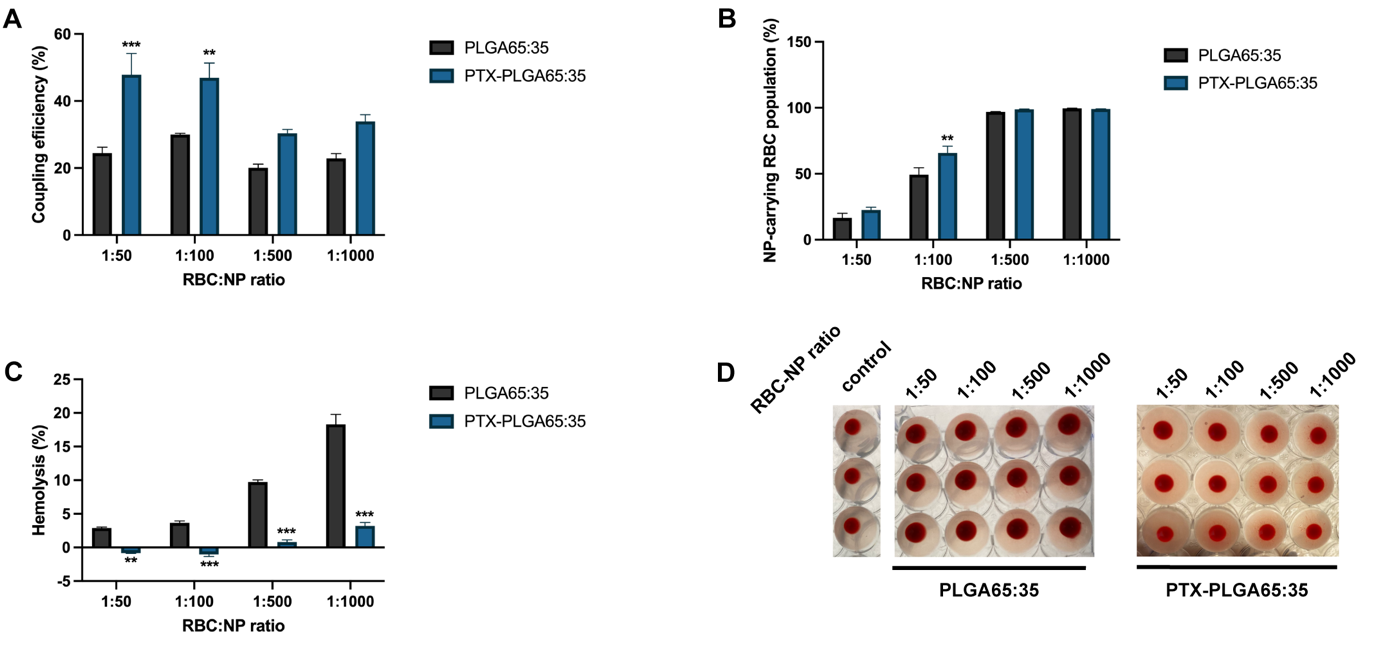


**Fig. S24** Effect of PTX encapsulation on NP adsorption onto human RBCs. (**a**) Coupling efficiency of PLGA65:35 and PTX-PLGA65:35 nanoparticles onto human RBCs at different RBC:NP incubation ratios. (**b**) Percentages of human RBC populations carrying PLGA65:35 or PTX-PLGA65:35 NP. (**c**) Hemolysis percentages of human RBCs after incubation with PLGA65:35 or PTX-PLGA65:35 NPs at different RBC:NP incubation ratios. Hemolysis percentages are represented as total hemolysis subtracted by hemolysis of control cells. (**d**) Agglutination assay of rabbit RBCs with PLGA65:35 or PTX-PLGA65:35 NPs at different RBC:NP incubation ratios (n=3). All data are presented as mean ± SEM (n = 3). The number of asterisks indicate the level of significance (two-way ANOVA with Sidak multiple testing adjustment), where: ** = p < 0.01 and *** = p < 0.001.
